# Developmental and Reproductive Impacts of *Arsenophonus* Symbiont on the Population of *Nilaparvata lugens*

**DOI:** 10.3390/insects17020222

**Published:** 2026-02-20

**Authors:** Qian-Qian Li, Salah M. Mohamed, Yi-Le Hu, Yong-Mao Lian, Adams Ibrahim, Xiang-Zhen Zhu, Feng Chen, Sheng Lin

**Affiliations:** 1 State Key Laboratory of Agricultural and Forestry Biosecurity, Fujian Agriculture and Forestry University, Fuzhou 350002, China; liqian654326@126.com (Q.-Q.L.); salah0038125@gmail.com (S.M.M.); hyl1067984243@163.com (Y.-L.H.); zhinao123258@163.com (Y.-M.L.); ibrahimadams682@gmail.com (A.I.); 2 International Joint Research Laboratory of Ecological Pest Control, Ministry of Education, Fujian Agriculture and Forestry University, Fuzhou 350002, China; 3 Ministerial and Provincial Joint Innovation Centre for Safety Production of Cross-Strait Crops, Fujian Agriculture and Forestry University, Fuzhou 350002, China; 4 Key Laboratory of Green Control of Insect Pests of Fujian Province, Fuzhou 350002, China; 5 Hui’an Carrot Science and Technology Courtyard, China Rural Special Technology Association, Quanzhou 362100, China; 6 Department of Pesticide & Toxicology, Faculty of Agricultural science, University of Gezira, Wad Madani 21111, Sudan; 7 State Key Laboratory of Cotton Biology, Institute of Cotton Research, Chinese Academy of Agricultural Science, Anyang 455000, China; 8 Institute of Plant Protection, Fujian Academy of Agricultural Sciences, Fuzhou 350013, China

**Keywords:** Delphacidae, gut microbiota, transgenic *cry30Fa1* rice, development, reproductive fitness, lipid metabolism

## Abstract

The Brown Planthopper, *Nilaparvata lugens* (Stål.) (Hemiptera: Delphinidae), is one of the most destructive pests of rice. Its reproductive and developmental traits are influenced by various environmental and biological factors including endosymbiotic microorganisms. *Arsenophonus*, a widespread endosymbiotic bacterium of insects, can affect host fitness and metabolic processes. This study investigates the role of *Arsenophonus* in modulating the developmental and reproductive traits of *N. lugens* fed on transgenic *cry30Fa1* rice (KF30-14) and its parent variety Minghui 86 (MH86). Life table analysis revealed that *Arsenophonus* infection (*Ars*^+^) increased the development time and reduced the reproductive capacity of *N. lugens*, especially those feeding on KF30-14. The first-instar nymphs in MH86 *Ars*^+^ (infected) exhibited slower development compared to MH86 *Ars^−^* (uninfected). Similarly, the third and fourth-instar nymphs in KF30-14 *Ars*^+^ exhibited prolonged development time compared to KF30-14 *Ars^−^*. In addition, KF30-14 *Ars*^+^ females had significantly reduced reproductive capacity, smaller ovarian tubules and lower relative expression levels of reproduction-related genes including *Trehalose transporter* (*Tret*), *Vitellogenin* (*Vg*) and *Cytochrome P450 hydroxylase* (*cyp314a1*), while J*uvenile hormone acid methyltransferase* (*JHAMT*) expression was upregulated. RNA sequencing and Kyoto Encyclopedia of Genes and Genomes (KEGG) pathway analysis revealed significant enrichment of genes involved in lipid, amino acid, and vitamin metabolisms, with *Long-chain acyl-CoA synthetase* implicated as a key regulator of lipid metabolism and reproductive fitness. These results highlight the complex interactions between endosymbionts, host plants and pest biology, offering a solid foundation for sustainable approaches to control *N. lugens* in rice production systems.

## Simple Summary

The Brown Planthopper (*Nilaparvata lugens)* is a major pest of rice that significantly impacts crop yields. This research explores how the bacterium *Arsenophonus*, a common microorganism in insects, influences the development and reproduction of *N. lugens*. The research compared the development and reproductive traits of *N*. *lugens* on two rice varieties: the transgenic variety KF30-14 and the non-transgenic Minghui 86. The results showed that *Arsenophonus* infection decreased the development of *N. lugens*, particularly when fed on the transgenic rice. Infected insects exhibited slower development and produced fewer offspring. Additionally, the infection altered the size of their reproductive organs and impacted important genes related to development and reproduction. These findings suggest that *Arsenophonus* can reduce the insect’s ability to reproduce, particularly on transgenic rice, potentially helping manage this insect in rice farming. This research highlights the complex relationship between insects’ microbial partners and their food sources. Understanding these interactions could lead to more effective and environmentally friendly insect control strategies in agriculture.

## 1. Introduction

The Brown planthopper *Nilaparvata lugens* (Stål.) (Hemiptera: Delphacidae) is an important pest that causes considerable crop losses, particularly in rice cultivation. Outbreaks result in yield losses of up to 80%, leading to annual losses of up to 300 million US dollars throughout Asia [1,2]. Control of this pest has become even more difficult with the emergence of pesticide resistant strains in East and Southeast Asia [3]. Consequently, alternative pest management strategies, such as genetic modifications in rice, have been explored, particularly through the development of transgenic rice varieties expressing *Bacillus thuringiensis* (Bt) proteins like *Cry30Fa1*. This *cry30Fa1* protein has insecticidal activity against Lepidoptera and Diptera insects, including the cabbage caterpillar (*Pieris rapae*) and mosquito species (*Aedes* spp). It is unclear whether *cry30Fa1* protein can provide desirable results in paddy fields [4].

The role of insect symbionts in shaping pest biology, including feeding behavior, reproductive capacity, and resistance mechanisms, has garnered increasing attention [5,6]. Insects, including *N. lugens*, rely heavily on their symbiotic bacteria to supplement their nutritional requirements, such as amino acids, vitamins, and sterols, which are deficient in their diet of plant phloem [7]. This interaction can influence the reproduction, development, and behavior of insects. For example, *Citrobacter* in the melon fruit fly (*Bactrocera dorsalis*) influences oviposition behavior and ovary development [8]. Olive fruit fly (*Bactroceraoleae*) without its natural microbiota has a reduced oviposition capacity [9]. The bacterium *Pseudomonas aeruginosa* shortens the lifespan of the Mediterranean fruit fly (*Ceratitis capitata*), while *Enterobacteriaceae* can prolong it [10,11]. In the case of rice stink bug (*Riptortus pedestris*), *Burkholderia* bacteria break down pesticides in the insect’s gut [12].

Among the diverse microbial communities associated with *N. lugens*, *Arsenophonus*, a genus of Enterobacteriaceae bacteria, plays a crucial role in modulating the host’s physiology, particularly reproductive success and lifespan. *Arsenophonus* is transmitted both vertically and horizontally within insect populations [13,14]. *Arsenophonus* affects lifespan, egg production, and hatchability of *N. lugens*, with region-specific effects on insect population dynamics [15]. Moreover, *Arsenophonus* increases resistance to fungal pathogens such as *Huanglingmyces* and may also influence resistance to insecticide [16]. Some *N. lugens* populations coexist with other symbionts such as *Wolbachia*, which modulate metabolic pathways associated with insecticide resistance [17]. For instance, *Serratia marcescens* plays a role in the metabolism of insecticides like imidacloprid and thiamethoxam in *N. lugens* [18]. In recent years, advances in insect genomics and transcriptomic technologies have greatly enhanced the understanding of pest biology at the molecular level [19]. High-throughput RNA sequencing (RNA-seq) has become a powerful tool for elucidating gene expression dynamics underlying insect development, metabolism, immunity, and stress responses to biotic and abiotic factors, including host plants, symbionts, pathogens, and insecticides [20]. This technique allows for a comprehensive evaluation of gene expression changes at a systems level, providing mechanistic insights that cannot be obtained from phenotypic observations alone. Specifically, RNA-seq enables the identification of key regulatory pathways involved in important biological traits, such as reproduction, metabolic processes, and development [21,22]. Importantly, in this study, RNA-seq was used to identify significant enrichment pathways related to lipid metabolism, amino acid metabolism, and vitamin metabolism, which are directly associated with phenotypic outcomes such as reproductive capacity and developmental timing. Differential Gene Expression (DEG) analysis and KEGG pathway enrichment were used to identify significant changes in gene expression that correlate with the observed phenotypic outcomes, such as reduced fecundity, developmental delays, and altered reproductive organ size [23]. By leveraging KEGG pathway analysis, we were able to link the altered expression of specific related genes to reproductive and metabolic changes, offering a comprehensive understanding of the molecular basis for the observed effects. These findings help explain the underlying molecular mechanisms driving the observed changes in *N. lugens* due to *Arsenophonus* infection. In addition, this study investigates the role of the symbiotic bacterium *Arsenophonus* in modulating the developmental and reproductive traits of *N. lugens* when fed on transgenic *Cry30Fa1* rice (KF30-14) and its parent variety, *Minghui 86*. The aim is to explore how this bacterium influences host physiology in relation to genetically modified crops, thereby providing insights into potential pest management strategies.

## 2. Materials and Methods

### 2.1. Rice Varieties and Insects Rearing

Two rice varieties, the insect-resistant transgenic rice (KF30-14) and its parent variety, Minghui 86 (MH86), were used in this study. Seeds were provided by the Key Laboratory of Agricultural Genetic Engineering, Fujian Academy of Agricultural Sciences. The rice was cultivated in a controlled greenhouse at the Institute of Applied Ecology, Fujian Agriculture and Forestry University, for 35 days, under stable conditions (26 ± 1 °C, 16:8 light/dark photoperiods, 80% ± 1% RH). *N. lugens* colony was obtained from the Institute of Virology, Fujian Agriculture and Forestry University, and was maintained for over 30 generations in an artificial climate chamber at the Institute of Applied Ecology.

### 2.2. Establishment of N. lugens Infected Populations and Bacterial Detection

In this study, the *Arsenophonus*-negative lines were experimentally established, and antibiotic treatment was used to eliminate *Arsenophonus* from the insect populations. The process involved exposing *N. lugens* to rice seedlings treated with 25 μg/mL ampicillin. This antibiotic was chosen based on its effectiveness in disrupting the gut microbiota of the insect, which was confirmed through previous studies [24,25,26]. The rice varieties (MH86 and KF30-14) were used to differentiate between transgenic Bt rice (KF30-14) and the non-transgenic control (MH86), while ampicillin exposure was employed to eliminate *Arsenophonus*. To ensure the antibiotic’s effectiveness in eliminating the symbiont, fifth-instar nymphs from each rice variety were transferred onto ampicillin-treated rice seedlings and reared for five consecutive generations. Antibiotic treatment was applied by exposing rice seedlings to ampicillin, after which the nymphs were maintained on treated plants throughout development. This protocol ensured continuous antibiotic exposure across generations, resulting in consistent disruption of the gut microbiota. After five generations, fifth-instar nymphs from laboratory colonies maintained on ampicillin-treated rice seedlings were selected, individually transferred to glass test tubes (15 cm × 2.5 cm), and reared to adulthood. Emerged adults (one male and one female per pair) were transferred to new tubes for mating. At the end of a one-week pairing period, females were collected for further analysis. Intestinal tissues were not dissected for structural examination; instead, whole-insect homogenates were used for DNA extraction and subsequent PCR analysis. DNA was extracted using the Fast Pure Cell/Tissue DNA Isolation Mini Kit (Nanjing NoVo Gene Bio-tech Co., Ltd., Nanjing, China) following the manufacturer’s protocol. The extracted DNA served as a template for PCR detection using *Arsenophonus*-specific primers (Table S1) and Phanta Max high-fidelity polymerase. The PCR products were then checked by agarose gel electrophoresis, with positive bands purified, sequenced, and confirmed by BLAST comparison against the NCBI database (https://www.ncbi.nlm.nih.gov/).

The *Arsenophonus* infection status of females and their eggs was determined and confirmed by PCR analysis. Based on these results, *Arsenophonus*-positive (*Ars^+^*) and *Arsenophonus*-negative (*Ars^−^*) lines were established and maintained on their respective rice varieties. Offspring (F1 generation) from 7*N. lugens Ars*^+^ and 7 *N. lugens Ars*^–^ were used to establish the colonies as experimental materials. Following antibiotic removal, no re-acquisition of *Arsenophonus* was observed in negative lines, confirming permanent elimination. Positive and negative lines were maintained separately under controlled laboratory conditions (26 ± 1 °C, 16L:8D photoperiod, 80% ± 1% RH) to prevent cross-contamination and ensure stable infection status; only confirmed individuals were used in subsequent experiments [27,28]. More details related to the phylogenetic analysis of *Ars* with simple diagram that explain this step clearly are provided in the Supplementary Materials (Figures S1, S2, and S3). This was a critical step to ensure that any observed differences in *N. lugens* development and reproduction were directly attributed to the presence or absence of *Ars* infection.

### 2.3. Treatment Groups and Design

Four different treatments were applied based on the presence and absence of *Arsenophonus* as follows: (i) MH86 *Ars*^−^ (MH86, *Arsenophonus*-negative); (ii) MH86 *Ars*^+^ (MH86, *Arsenophonus*-positive); (iii) KF30-14 *Ars*^−^ (KF30-14, *Arsenophonus*-negative); (iv) KF30-14 *Ars*^+^ (KF30-14, *Arsenophonus*-positive). MH86 *Ars^−^* served as the baseline control, whereas comparisons between the *Ars^+^* and *Ars*^−^ treatments within each rice variety were used to assess the impact of *Arsenophonus*.

### 2.4. Life Table Analysis

To determine the effect of *Arsenophonus* infection on the population demography of *N. lugens* reared under the four treatments. Five pairs from each treatment were used with 90 individuals per treatment for life table recording. The developmental and reproductive parameters of *N. lugens* of the four treatments (MH86 *Ars^−^*, MH86 *Ars^+^,* KF30-14 *Ars^−^*, and KF30-14 *Ars^+^*) were analyzed with 5th generation. For each population, the duration of nymphal instars (1st–5th) and the total developmental period were recorded. Survival rates at all life stages, including nymphs and adults, were recorded. Age-specific fecundity (*f*_x_), net reproductive rate (*m*_x_), and population-specific net reproductive rate (*l*_x_*m*_x_) were calculated to assess reproductive capacity. Egg-laying capacity was monitored daily for each group, and reproductive data, including the number of eggs laid by females and age-stage-specific reproductive values (*v*_xj_), where *x* represents age and *j* represents stage, were recorded. The intrinsic growth rate, generation time, and population fitness were calculated from the life table data (Table S2). These data are critical for assessing the demographic effects of *Ars* infection and its potential implications for pest management strategies in the presence of this transgenic or non-transgenic rice.

### 2.5. Effect of Ars on Adult Weight and the Size of the Reproductive Organs of *N. lugens* Fed on Different Rice Varieties

The fifth-instar nymphs from four treatments (MH86 *Ars*^−^, MH86 *Ars*^+^, KF30-14 *Ars*^−^, and KF30-14 *Ars*^+^) were individually reared in test tubes. The emergence of *N. lugens* was observed daily. The measurements of adult weight and newly emerged male and female adults were collected daily after emergence. For each replicate, 10 individuals were placed into a centrifuge tube and weighed. The size of the reproductive organs was measured using newly emerged adults, collected 1 day and 3 days after emergence. The collected *N. lugens* were dissected under a stereomicroscope, and the sizes of the reproductive organs were measured using a measurement system in the software. The average of the two sides of the reproductive organs was taken for analysis. For both adult weight and reproductive organ size measurements, 10 insects were used per replicate, with three biological replicates for each treatment.

### 2.6. Gene Expression and Transcriptomic Analysis

#### 2.6.1. RNA Extraction

For gene expression analysis, individual newly emerged females of *N. lugens* were collected from each treatment group (MH86 *Ars^−^*, MH86 *Ars^+^*, KF30-14 *Ars^−^*, and KF30-14 *Ars*^+^). RNA extraction was performed on each sample; for each replicate, 10 newly emerged female adults were collected, with three biological replicates. These insects were quickly frozen in liquid nitrogen and stored at −80 °C until RNA extraction. Total RNA was isolated using the RNAprep Pure Insect Kit (Tiangen, Beijing, China) according to the manufacturer’s instructions. RNA quality was assessed using a NanoDrop spectrophotometer, and agarose gel electrophoresis was assessed for integrity. For the RNA quality details, see Supplementary Materials Table S4.

#### 2.6.2. Real-Time Quantitative PCR (qPCR)

To evaluate gene expression, the following reproductive-related genes were selected and analyzed: vitellogenin (*Vg*), juvenile hormone acid methyltransferase (*JHAMT*), cytochrome P450 hydroxylase (*cyp314a1*), and trehalose transporter (*Tret*). These genes are essential for energy supply during development and reproduction and thus provide molecular insight into how Ars infection might modulate host physiology. The expression levels of these genes were measured using qPCR. The β-actin gene was used as an internal control. The primer sequences used for qPCR are provided in Table S3.

#### 2.6.3. Transcriptome Data Processing and Quality Control

For transcriptomic analysis, newly emerged female adults of *N. lugens* were used as the biological material. Fifth-instar nymphs from four treatments (MH86 *Ars*^−^, MH86 *Ars^+^*, KF30-14 *Ars*^−^, and KF30-14 *Ars^+^*) were first transferred onto fresh rice seedlings and reared individually until adult emergence. Newly emerged female adults were then collected to ensure developmental consistency and to minimize variation caused by sex and age. For each treatment, three independent biological replicates were prepared, with 40 female adults per replicate. Collected insects were rapidly frozen in liquid nitrogen for 30 min and stored at −80 °C until RNA extraction. Total RNA was extracted using a commercial RNA extraction kit as mentioned previously, and only samples meeting quality standards were used for downstream transcriptome sequencing. These RNA samples were subsequently sent to a professional sequencing company for Illumina-based transcriptome sequencing.

#### 2.6.4. Differential Gene Expression and KEGG Enrichment Analysis

Differential gene expression (DEG) analysis was performed using the DESeq2 package (version 1.28.1), with a cutoff for adjusted *p*-value set at ≤ 0.05. Differential expressions were evaluated for the following comparisons: MH86 *Ars^−^* vs. MH86 *Ars*^+^, KF30-14 *Ars^−^* vs. KF30-14 *Ars*^+^, MH86 *Ars^−^* vs. KF30-14 *Ars^−^*, and MH86 *Ars*^+^ vs. KF30-14 *Ars*^+^*,* and Kyoto Encyclopedia of Genes and Genomes (KEGG) pathway analyses were conducted. These analyses were performed using Blast2GO and KOBAS 3.0 to identify biological processes and pathways affected by *Ars. The* KEGG pathway, which focused on pathways associated with fatty acid metabolism and apoptosis, was conducted to explore potential links to reproductive changes in *N. lugens* feeding on different rice varieties. Pathway visualization was performed using the Cluster Profiler R package.

### 2.7. Statistical Analysis

The statistical analysis was performed on samples of sequence data using various software tools. Life table parameters were analyzed using TWOSEX-MSChart software (Ver: 1/25/2026) [29]. Bootstrap resampling (100,000 iterations) was used to estimate variability, the Timing program [30] was used to predict the population dynamics of the *N. lugens* over the next 60 days, and Sigma Plot 14.0 was used for graphing. The relative gene expression levels were calculated using the 2^-^ΔΔCt method [31], and SPSS 22.0 software was used for analysis of the weight and genital size data. A one-way ANOVA was performed to compare the means of different treatment groups, followed by Duncan’s multiple range test to assess significant differences between treatments, considered statistically significant at *p* < 0.05. Graphs were generated using GraphPad Prism 9.0.

## 3. Results

### 3.1. Establishment and Confirmation of Ars^−^ Infected Populations

**Figure 1:**
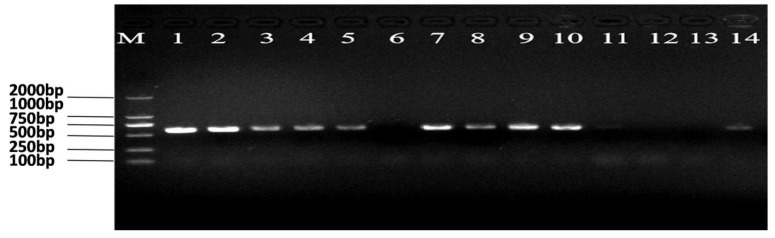
Confirmation of *Arsenophonus* infection in *Nilaparvata lugens* colonies. (M) DNA marker DL2000; Lanes 1–7, *Nilaparvata lugens* fed on KF30-14; Lanes 8–14, *Nilaparvata lugens* fed on MH86. Numbers with the bright band are representing the presence and the case of *Arsenophonus* infection.

Based on the established methodology described above, reciprocal crossing experiments between *Ars*^+^ and *Ars*^−^ adults demonstrated that infection was inherited exclusively through the maternal line, confirming that *Ars* is vertically transmitted via the eggs. PCR amplification produced distinct bands corresponding to the expected amplicon size, indicating the presence of *Ars*. The identity of the amplified fragment was further confirmed by sequencing and BLAST analysis against the NCBI database. Accordingly, both *Ars*^+^ and *Ars*^−^ populations of *N. lugens* were successfully established as described above, and their infection status is illustrated by the PCR gel image (Figure 1).

### 3.2. Effect of Ars on the Fitness of *N. lugens* Populations Fed on Different Rice Varieties

**Figure 2:**
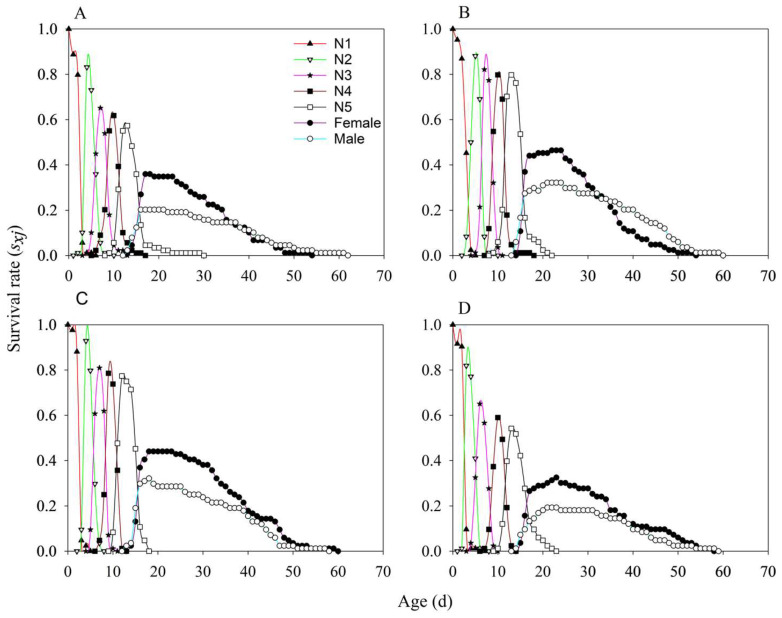
Survival rates of *Nilaparvata lugens* nymphs and adults in different *Arsenophonus* treatments. (**A**) *Nilaparvata lugens* without *Arsenophonus* fed on MH86 (MH86 *Ars^−^*); (**B**) *Nilaparvata lugens* with *Arsenophonus* fed on MH86 (MH86 *Ars*^+^); (**C**) *Nilaparvata lugens* without *Arsenophonus* fed on KF30-14 (KF30-14 *Ars^−^*); (**D**) *Nilaparvata lugens* with *Arsenophonus* fed on KF30-14 (KF30-14 *Ars*^+^).

**Figure 3:**
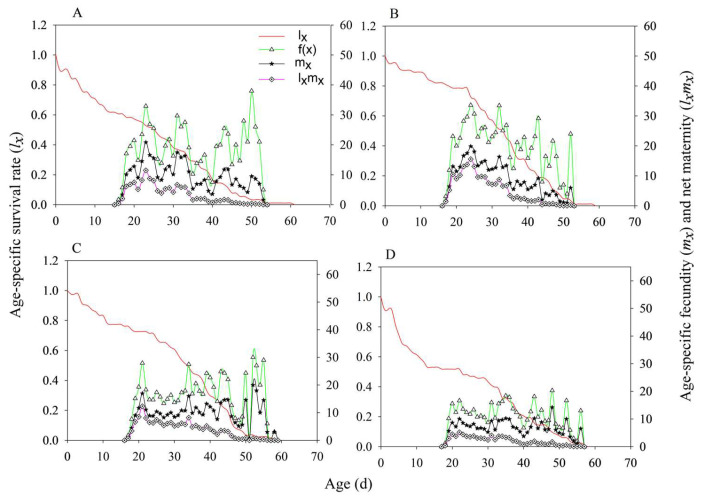
Age-specific net maternity of *Nilaparvata lugens* (*l_x_m_x_*) under different treatments. (**A**) *Nilaparvata lugens* without *Arsenophonus* fed on MH86 (MH86 *Ars^−^*); (**B**) *Nilaparvata lugens* with *Arsenophonus* fed on MH86 (MH86 *Ars*^+^); (**C**) *Nilaparvata lugens* without *Arsenophonus* fed on KF30-14 (KF30-14 *Ars^−^*); (**D**) *Nilaparvata lugens* with *Arsenophonus* fed on KF30-14 (KF30-14 *Ars*^+^).

**Figure 4:**
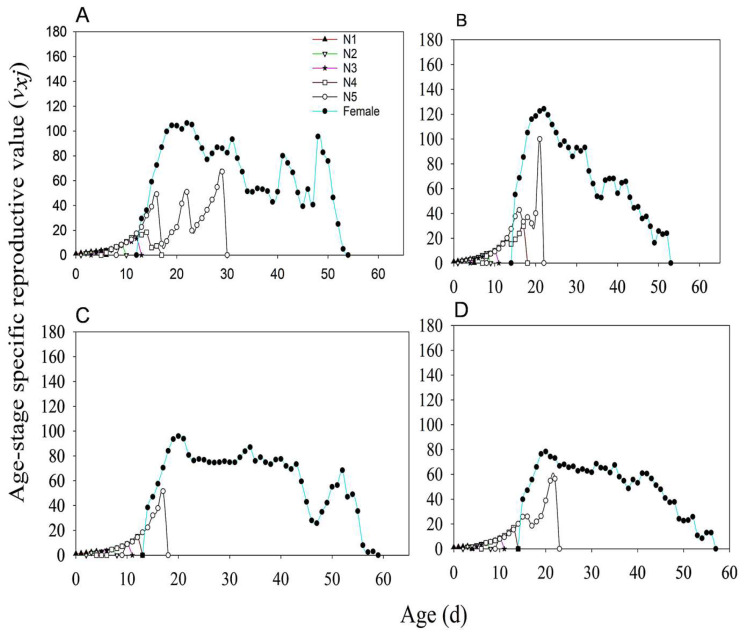
Age-stage specific reproductive values of *Nilaparvata lugens* under different treatments. (**A**) *Nilaparvata lugens* without *Arsenophonus* fed on MH86 (MH86 *Ars^−^*); (**B**) *Nilaparvata lugens* with *Arsenophonus* fed on MH86 (MH86 *Ars*^+^); (**C**) *Nilaparvata lugens* without *Arsenophonus* fed on KF30-14 (KF30-14 *Ars^−^*); (**D**) *Nilaparvata lugens* with *Arsenophonus* fed on KF30-14 (KF30-14 *Ars*^+^).

The survival rate curves of *N. lugens* under the four treatments (MH86 *Ars*^−^, MH86 *Ars*^+^, KF30-14 *Ars*^−^, and KF30-14 *Ars*^+^) showed considerable overlap, with the age–stage-specific survival rate (*sₓⱼ*) of nymphs being consistently higher than that of adults (Figure 2). The *sₓⱼ* values of 3rd, 4th, and 5th-instar nymphs in MH86 *Ars*^−^ were lower than those in MH86 *Ars*^+^ (Figure 2A,B), whereas in KF30-14 *Ars*^−^, the *sₓⱼ* values of the 3rd, 4th, and 5th-instar nymphs were higher than those in KF30-14 *Ars*^+^ (Figure 2C,D). Before 35 days, the *sₓⱼ* of females was higher than that of males across all four treatments (Figure 2). For reproductive capacity of female adults, particularly in MH86 *Ars^−^*, the age-stage-specific fecundity (*f*_x_) reached its peak of 38.00 at day 50, while *f*_x_ in MH86 *Ars***^+^** had its peak (33.59) at day 24. Compared to KF30-14 *Ars^−^*, *f*_x_ reached a value of 30.00 on day 52, whereas in KF30-14 *Ars***^+^** it reached a value of 18.11 on day 43. The net reproduction rate (*m_x_*) and the population-specific net reproduction rate (*l*_x_*m*_x_) were higher in MH86 *Ars^−^* compared to MH86 *Ars***^+^ (**Figure 3A,B), while the opposite was true for KF30-14 *Ars^−^* and KF30-14 *Ars***^+^**(Figure 3C,D). The initial reproductive values of MH86 *Ars*^−^, MH86 *Ars*^+^, KF30-14 *Ars*^−^, and KF30-14 *Ars*^+^ were 29.49, 55.23, 38.44, and 39.89, respectively. In all four treatments, the reproductive value of *N. lugens* increased with age and developmental stage, reaching its maximum during the adult stage, indicating that adults made the greatest contribution to future population growth (Figure 4). The age-stage-specific reproductive value (*vₓⱼ*) of MH86 *Ars*^−^ peaked on day 22 at 106.43, whereas MH86 *Ars*^+^ reached its maximum (124.32) on the same day (Figure 4A,B). KF30-14 *Ars*^−^ and KF30-14 *Ars*^+^ peaked on day 20 with *vₓⱼ* values of 95.93 and 78.38, respectively (Figure 4C,D).

**Figure 5:**
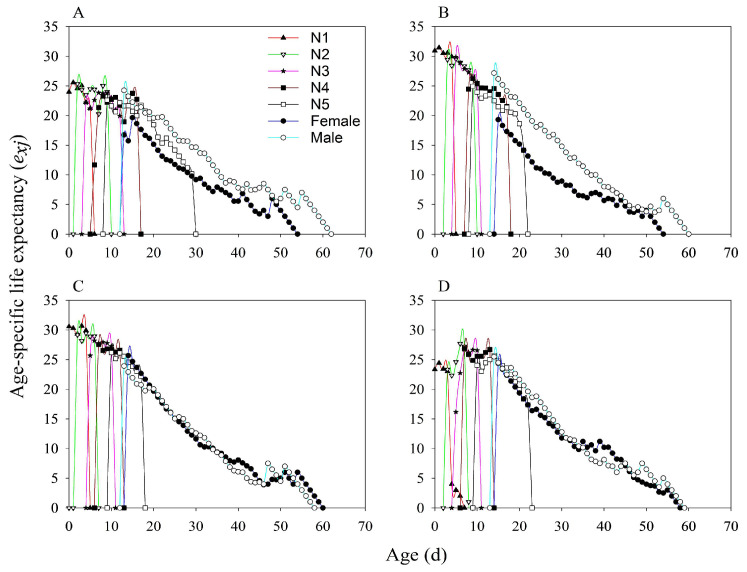
Age-stage specific life expectancy of *Nilaparvata lugens* under different treatments. (**A**) *Nilaparvata lugens* without *Arsenophonus* fed on MH86 (MH86 *Ars^−^*); (**B**) *Nilaparvata lugens* with *Arsenophonus* fed on MH86 (MH86 *Ars*^+^); (**C**) *Nilaparvata lugens* without *Arsenophonus* fed on KF30-14 (KF30-14 *Ars^−^*); (**D**) *Nilaparvata lugens* with *Arsenophonus* fed on KF30-14 (KF30-14 *Ars*^+^).

The age-stage-specific life expectancy of *N. lugens* populations reared on both the MH86 and KF30-14 rice varieties generally decreased as the insects aged (Figure 5). In the MH86 treatments, the life expectancy of 1st, 2nd, and 3rd-instar nymphs was lower in MH86 *Ars*^−^ than in MH86 *Ars*^+^. The life expectancy of 3rd instar nymphs in MH86 *Ars*^−^ showed a pattern of decline followed by a partial recovery, indicating a higher mortality rate at this stage (Figure 5A,B). In contrast, the 1st, 2nd, and 3rd-instar nymphs of KF30-14 *Ars*^−^ exhibited higher life expectancy values than those of KF30-14 *Ars*^+^. The life expectancy of 2nd-instar nymphs in KF30-14 *Ars*^−^ also showed a decrease followed by an increase, suggesting elevated mortality during this stage (Figure 5C,D).

### 3.3. Stable Age-Stage Distribution of Nymphs (SASD)

**Figure 6:**
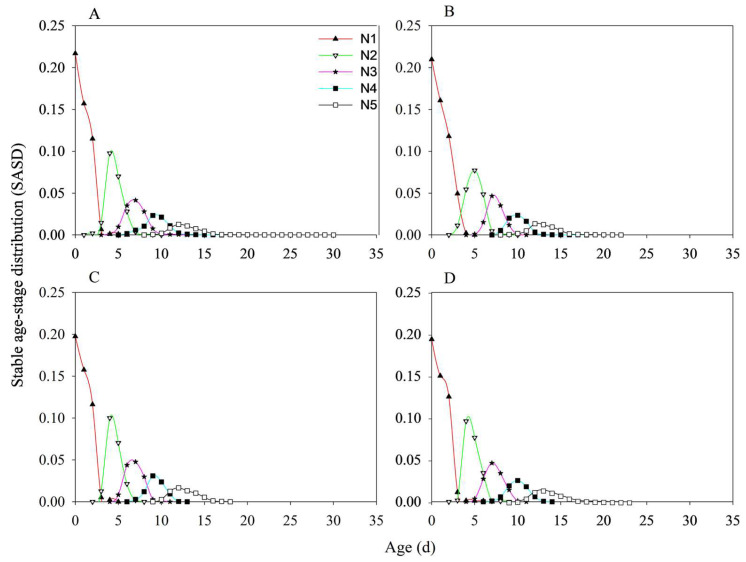
The pre-adult stable age-stage distribution of *Nilaparvata lugens* nymphs. (**A**) *Nilaparvata lugens* without *Arsenophonus* fed on MH86 (MH86 *Ars^−^*); (**B**) *Nilaparvata lugens* with *Arsenophonus* fed on MH86 (MH86 *Ars*^+^); (**C**) *Nilaparvata lugens* without *Arsenophonus* fed on KF30-14 (KF30-14 *Ars^−^*); (**D**) *Nilaparvata lugens* with *Arsenophonus* feeding on KF30-14 (KF30-14 *Ars*^+^).

The stable age-stage distributions (SASD) of nymphs under different treatments are shown in Figure 6. The proportions of 1st and 2nd instar nymphs were higher in MH86 *Ars*^−^ than in MH86 *Ars*^+^, whereas the duration of the 1st instar nymphs were shorter and that of the 5th instar nymphs were longer in MH86 *Ars*^−^ than in MH86 *Ars*^+^ (Figure 6A,B). The 5th-instar nymphs of KF30-14 *Ars*^+^ exhibited longer survival than those of KF30-14 *Ars*^−^ (Figure 6C,D). Compared with KF30-14 *Ars*^−^, MH86 *Ars*^−^ had a higher SASD for 1st-instar nymphs and longer survival during the 4th and 5th-instar nymphs (Figure 6A,C). Similarly, MH86 *Ars*^+^ showed a higher SASD for 1st-instar nymphs but a lower SASD for 2nd-instar nymphs than KF30-14 *Ars*^+^ (Figure 6B,D). 

### 3.4. Stable Age-Stage Distribution of Adults (SASD)

**Figure 7:**
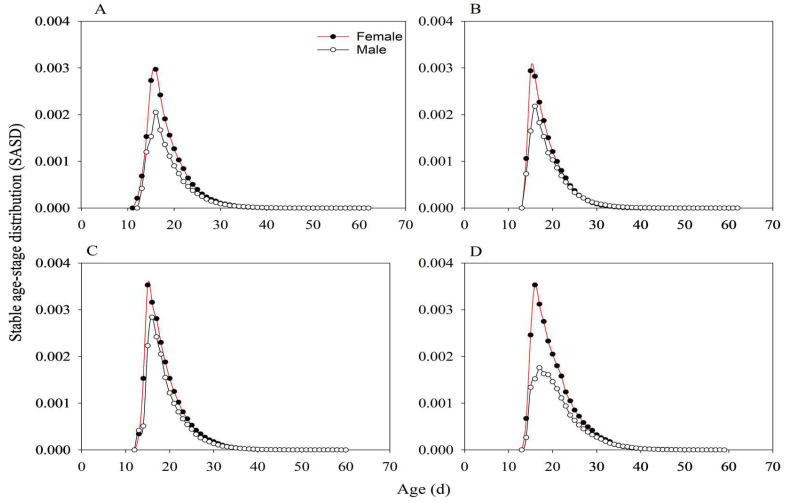
Adult stable age-stage distribution of *Nilaparvata lugens* (SASD). (**A**) *Nilaparvata lugens* without *Arsenophonus* fed on MH86 (MH86 *Ars^−^*); (**B**) *Nilaparvata lugens* with *Arsenophonus* fed on MH86 (MH86 *Ars*^+^); (**C**) *Nilaparvata lugens* without *Arsenophonus* fed on KF30-14 (KF30-14 *Ars^−^*); (**D**) *Nilaparvata lugens* with *Arsenophonus* fed on KF30-14 (KF30-14 *Ars*^+^).

For adults under different treatments, presented in Figure 7. The SASD of male adults in KF30-14 *Ars*^−^ was higher than that in KF30-14 *Ars*^+^ (Figure 7C,D). Both female and male adults of MH86 *Ars*^−^ had lower SASD values than those of KF30-14 *Ars*^−^ (Figure 7A,C). In contrast, the SASD of female adults in MH86 *Ars*^+^ was lower than that in KF30-14 *Ars*^+^, whereas the SASD of male adults in MH86 *Ars*^+^ was higher than that in KF30-14 *Ars*^+^ (Figure 7B,D).

### 3.5. Developmental Duration and Lifespan of N. lugens

The development periods of the 1st, 3rd, and 4th instar nymphs were shorter in MH86 *Ars^−^* than in MH86 *Ars*^+^. In contrast, KF30-14 *Ars^−^* had shorter development periods for the 3rd and 4th instars than KF30-14 *Ars*^+^. The 3rd and 4th instar nymphs, adult pre-oviposition period (APOP), and total pre-oviposition period (TPOP) of KF30-14 *Ars*^+^ were higher than those of KF30-14 *Ars^−^*, while the total lifespan and fecundity of KF30-14 *Ars*^+^ were lower than those of KF30-14 *Ars^−^*. The developmental duration of the 3rd and 5th instar nymphs and the adult female longevity of KF30-14 *Ars*^+^ were higher than those of MH86 *Ars*^+^, whereas the fecundity of KF30-14 *Ars*^+^ was lower than that of MH86 *Ars*^+^ (Table 1 and Table 2).

### 3.6. Mortality Rate Distribution

The mortality rates differed significantly (*p* < 0.05); for the 3rd and 4th instars, they were higher in MH86 *Ars^−^* than in MH86 *Ars***^+^**, while KF30-14 *Ars^−^* had lower mortality rates during the nymphal stage than KF30-14 *Ars***^+^** (Table 3).

### 3.7. Population Parameters and Dynamics Predication

**Figure 8:**
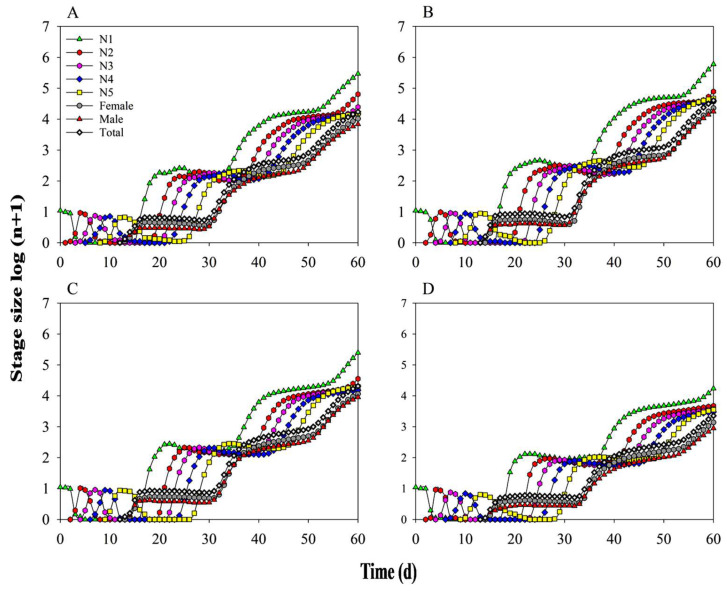
Predicted population growth of *Nilaparvata lugens* under different treatments. (**A**) *Nilaparvata lugens* without *Arsenophonus* fed on MH86 (MH86 *Ars^−^*); (**B**) *Nilaparvata lugens* with *Arsenophonus* fed on MH86 (MH86 *Ars*^+^); (**C**) *Nilaparvata lugens* without *Arsenophonus* fed on KF30-14 (KF30-14 *Ars^−^*); (**D**) *Nilaparvata lugens* with *Arsenophonus* fed on KF30-14 (KF30-14 *Ars*^+^).

Population parameters including intrinsic growth rate, net reproductive rate, and mean generation time, were compared to evaluate the effects of diet and bacterial presence on *N. lugens*. The intrinsic growth rate was significantly higher in KF30-14 *Ars^−^* than in KF30-14 *Ars*^+^, while MH86 *Ars*^+^ exhibited a significantly higher intrinsic growth rate than KF30-14 *Ars*^+^ (Table 4). A 60-day population growth simulation further predicted higher population sizes for MH86 *Ars*^−^, MH86 *Ars*^+^, and KF30-14 *Ars*^−^, whereas KF30-14 *Ars*^+^ exhibited the lowest predicted population growth (Figure 8).

### 3.8. Effect of Arsenophonus on Adult Weight

**Figure 9:**
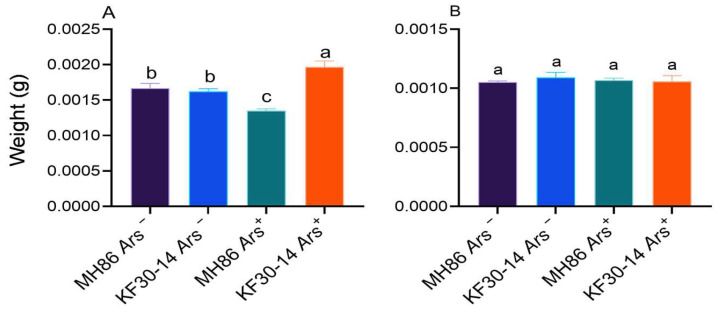
Weight of first emergence adult *Nilaparvata lugens.* (**A**) Body weight of adult females; (**B**) Body weight of adult males. Different lowercase letters indicated significant differences between treatments. Statistical analysis was conducted using one-way ANOVA to compare the means of different treatment groups, followed by Duncan’s multiple range test to assess significant differences between treatments, considered statistically significant at *p* < 0.05.

Adult females of MH86 *Ars***^+^** exhibited significantly lower body weight than those on MH86 *Ars^−^*, whereas adult females of KF30-14 *Ars^+^* showed significantly higher body weight compared with KF30-14 *Ars^−^* (Figure 9A). In contrast, no significant differences in adult male body weight were detected among treatments (Figure 9B).

### 3.9. Effect of Arsenophonus on the Size of the Reproductive Organs of *N. lugens* Feeding on Different Rice Varieties

**Figure 10:**
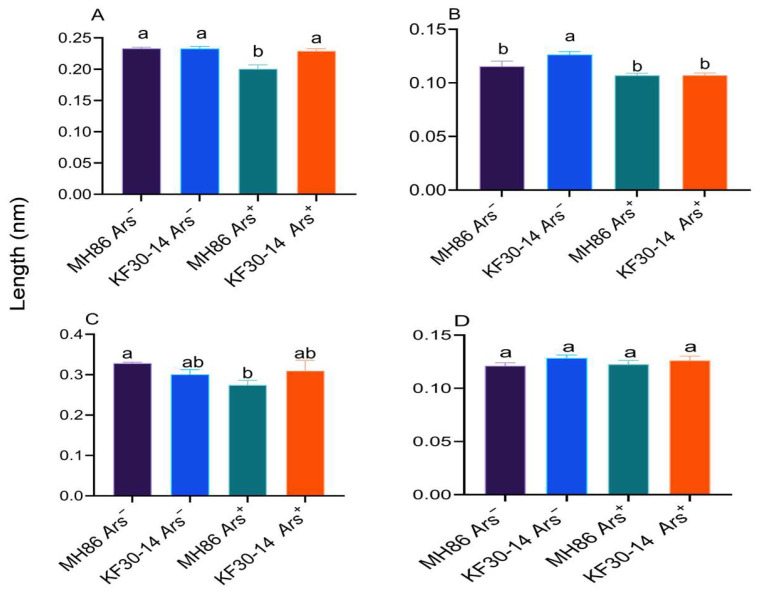
Genital size of *Nilaparvata lugens* under different *Arsenophonus* infection statuses on two rice varieties. (**A**) ovariole length of newly emerging female adult; (**B**) testis length of newly emerging male adults; (**C**) ovariole length of female adult after emergence for three days; (**D**) testis length of male adults after emergence for three days. Different case letters indicated significant differences between treatments. Statistical analysis was conducted using one-way ANOVA to compare the means of different treatment groups, followed by Duncan’s multiple range test to assess significant differences between treatments, considered statistically significant at *p* < 0.05.

The length of ovaries and testes was affected by both *Arsenophonus* infection and rice variety. For ovarian tubule length of newly emerged female adults, no significant difference was found between MH86 *Ars^−^* and KF30-14 *Ars^−^*, whereas MH86 *Ars*^+^ had significantly shorter ovarian tubules than KF30-14 *Ars*^+^ (Figure 10A). In MH86, ovarian tubule length in female adults three days after emergence was significantly longer in *Ars^−^* than in *Ars^+^*, while no significant difference was observed between KF30-14 *Ars^−^* and KF30-14 *Ars^+^* (Figure 10C). For testis length in newly emerged male adults, MH86 *Ars*^−^ individuals had significantly shorter testes than KF30-14 *Ars*^−^, whereas no significant difference was found between MH86 *Ars*^+^ and KF30-14 *Ars*^+^; in both rice varieties, *Arsenophonus* infection reduced testis length (Figure 10B), while for testis length of male adults three days after emergence, no significant difference was found among all treatments (Figure 10D). These findings suggest that reproductive organ size is affected by both rice variety and bacterial infection, with MH86 *Ars^−^* individuals having longer ovarian tubules, while testicle length varied depending on the host plant.

### 3.10. Effect of Arsenophonus Infection on the Expression of Reproductive Genes in Newly Eclosed Female Adults

**Figure 11:**
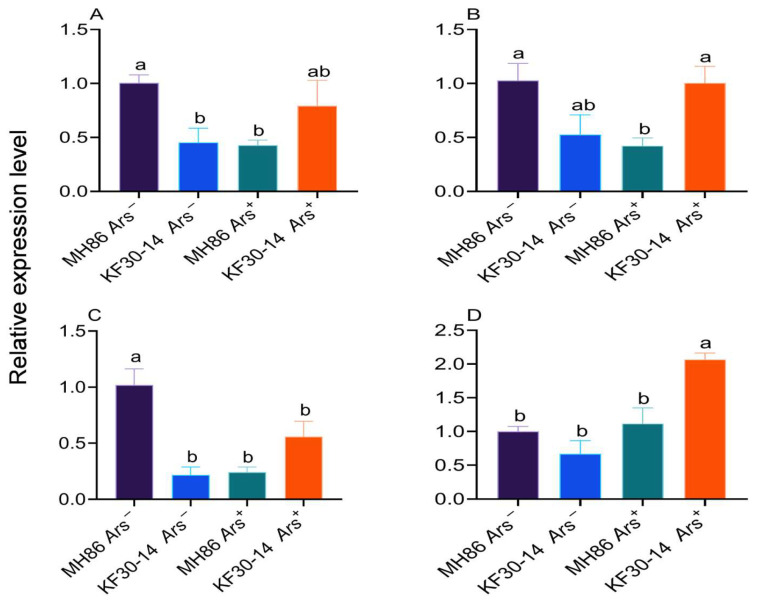
Relative expression of reproductive-related genes in newly eclosed female *Nilaparvata lugens*. (**A**) Trehalose transporter (*Tret*); (**B**) Vitellogenin (*Vg*); (**C**) Cytochrome P450 hydroxylase (*cyp314a1*); (**D**) Juvenile hormone acid methyltransferase (*JHAMT*). Different case letters indicated significant differences between treatments. Statistical analysis was conducted using one-way ANOVA to compare the means of different treatment groups, followed by Duncan’s multiple range test to assess significant differences between treatments, considered statistically significant at *p* < 0.05.

The expression of the reproduction-related genes *Tret, Vg, cyp314a1,* and *JHAMP* was analyzed in newly eclosed female *N. lugens* in MH86 and KF30-14 rice varieties. The expression level of the *Tret gene* was significantly higher in MH86 *Ars^−^* than in KF30-14 *Ars^−^*, whereas it was lower in MH86 *Ars*^+^ than in KF30-14 *Ars*^+^ (Figure 11A). A similar pattern was observed for the *Vg* expression levels (Figure 11B). The expression of *cyp314a1* was significantly higher in MH86 *Ars^−^* than KF30-14*Ars^−^*; however, no significant differences were detected between MH86*Ars*^+^ and KF30-14*Ars*^+^ (Figure 11C). In contrast, *JHAMP* expression remained unchanged among most treatments, but KF30-14 *Ars*^+^ exhibited a significantly higher expression level compared with all other treatments (Figure 11D). These findings indicate that *Ars* infection alters the gene expression of reproductive organs, particularly in MH86, with reduced *Tret, Vg,* and *cyp314a1* expressions and increased *JHAMP* expression in KF30-14 *Ars*^+^. 

### 3.11. Transcriptome Sequencing Quality and Differential Expression Analysis

High-quality transcriptome sequencing data were obtained across all samples. After filtering, clean reads showed stable GC content and high sequencing quality, with mapping rates ranging from 80.23% to 82.69%, indicating good alignment to the *N. lugens* reference genome and minimal contamination (Tables S5 and S6; Figure S4). Differentially expressed genes (DEGs) among treatments were clearly visualized using volcano plots and hierarchical clustering heat maps, revealing distinct expression patterns associated with *Ars* status and rice diet (Figures S6 and S7A–F). Functional annotation of DEGs using Gene Ontology (GO) analysis showed enrichment in biological processes related to metabolism, cellular processes, and immune responses (Figure S5). KEGG pathway enrichment further indicated that DEGs were significantly involved in pathways associated with metabolic regulation, signal transduction, and host defense mechanisms (Figures S8 and S9), highlighting the molecular responses of *N. lugens* to symbiont presence under different rice treatments.

#### 3.11.1. KEGG Annotation Analysis

KEGG pathway enrichment analysis identified distinct biological pathways across different treatments, including metabolism, genetic information processing, and environmental information processing. MH86 *Ars*^+^ was enriched in purine metabolism, retinol metabolism, glycerolipid metabolism, and fat digestion compared to MH86 *Ars^−^*. In KF30-14 *Ars*^+^, the key enriched pathways were D-amino acid metabolism, proximal tubule bicarbonate reclamation, and arginine biosynthesis. Comparison of MH86 *Ars^−^* and KF30-14 *Ars^−^* rice varieties revealed enrichment in transport, catabolism, and lipid metabolism pathways, whereas comparing of MH86 *Ars*^+^ and KF30-14 *Ars*^+^ revealed enrichment in apoptosis-related pathways and neurodegeneration. Additionally, MH86 *Ars^−^* vs. KF30-14 *Ars*^+^ showed significant enrichment in signal transduction, lipid metabolism, and cancer-related pathways, whereas MH86 *Ars*^+^ vs. KF30-14 *Ars^−^* were enriched in fatty acid elongation, D-amino acid metabolism, and vitamin digestion. These findings suggest that both infection with *Ars* and the host plant genotype influence critical metabolic and regulatory pathways in *N. lugens* (Figure S10A–F).

#### 3.11.2. Differentially Expressed Genes Between Treatments and KEGG Enrichment Analysis

The comparison between KF30-14 Ars^−^ and KF30-14 Ars^+^ unveiled 373 differentially expressed genes (DEGs) mapped to 211 KEGG pathways, with the most represented categories being human diseases (58 pathways) and metabolism (54 pathways). KEGG enrichment analysis across six pairwise comparisons revealed distinct pathway patterns: MH86 Ars^−^ vs. MH86 Ars^+^ showed enrichment in purine, retinol, and glycerolipid metabolisms; KF30-14 Ars^−^ vs. KF30-14 Ars^+^ was enriched in D-amino acid metabolism and arginine biosynthesis; MH86 Ars^−^ vs. KF30-14 Ars^−^ showed enrichment in transport and catabolism; MH86 Ars^+^ vs. KF30-14 Ars^+^ in apoptosis and neurodegenerative diseases; MH86 Ars^−^ vs. KF30-14 Ars^+^ in signal transduction and lipid metabolism; and MH86 Ars^+^ vs. KF30-14 Ars^−^ in fatty acid elongation and vitamin digestion (Figures S11 and S12A–F).

## 4. Discussion

Symbiotic microorganisms in insects have a significant influence on various physiological processes, such as behavior, reproductive capacity, development, and life expectancy [32,33]. In *Drosophila melanogaster*, for example, symbiotic yeasts such as *Saccharomyces cerevisiae* and *Acetobacter malorum* have been shown to improve reproductive capacity, shorten larval development time, and increase ovary size, demonstrating the beneficial effects of these symbionts on *D*. *melanogaster* [34]. In the pea aphid (*Acyrthosiphon pisum*), the obligate symbiont *Buchnera aphidicola* plays a critical role in host nutrition by synthesizing essential amino acids that are deficient in the phloem sap diet; this symbiosis significantly enhances aphid growth, fecundity, and survival [35]. Disruption or loss of *Buchnera* results in reduced body size, delayed development, and decreased reproductive output, clearly demonstrating the dependence of aphid fitness on its microbial symbionts [36]. Similarly, in this study, *Arsenophonus* infection in *N. lugens* causes developmental delays and reduced reproduction, likely due to disruptions in lipid and amino acid metabolism. The impact of *Arsenophonus* is modulated by the host plant, highlighting the complex interplay between symbionts, host physiology, and environmental factors.

The study clarifies the role of the secondary symbiont *Ars* in shaping the fitness of *N. lugens* when feeding on various rice varieties, including both transgenic Bt rice and a non-transgenic control. Using life-table analyses, gut microbiota manipulation, and comparative assessments across rice treatments, we evaluated survival, development, and reproductive performance. The results found that *Ars* bacteria influence the survival rate of *N. lugens* fed on different rice varieties, consistent with findings in the tsetse fly (*Glossina morsitans*), where symbiont disruption reduced larval survival, highlighting the critical role of symbiotic bacteria in host physiology and fitness [37,38].

The contrasting effects of *Ars* on nymph survival across rice varieties indicate a strong host plant-microbe interaction. The lower nymph mortality observed in MH86 *Ars^+^* compared with MH86 *Ars^−^* suggests a potentially mutualistic role of *Ars* under non-Bt feeding conditions, consistent with reports showing symbiont-mediated fitness benefits in hemipteran insects [39]. In contrast, the increased nymph mortality and reduced fecundity in KF30-14 *Ars^+^* indicate that *Ars* becomes detrimental when insects feed on KF30-14 rice, likely due to synergistic stress between Bt toxins and symbiont-induced metabolic costs. The shorter generation time on MH86 compared with KF30-14 further suggests that Bt rice imposes developmental constraints, which may be exacerbated by symbiont presence. The adult female weight of *N. lugens* was lower on MH86 *Ars^+^*, while the opposite trend was observed on KF30-14 *Ars^+^*, where the weight of *N. lugens* significantly increased. This discrepancy could be caused by the interaction of *Cry* proteins in transgenic rice, which could induce stress. Symbiotic bacteria such as *Arsenophonus* can assist insects in managing stress by improving nutrient acquisition, metabolic compensation, or stress tolerance, resulting in increased weight on KF30-14; similar results have been observed in other studies of insect host adaptation [40].

These results suggest that infection with the bacterium *Ars*^+^ significantly affects the development and reproductive capacity of *N. lugens*, especially in populations feeding on the transgenic *cry30Fa1* rice variety (KF30-14). Infected first-instar nymphs of MH86 *Ars^+^* (non-transformed rice) showed a longer development time compared to their uninfected counterparts (MH86 *Ars^−^*); similarly, the development time for the 3rd and 4th-instar nymphs of KF30*-14 Ars^+^* was longer than that of KF30*-14 Ars^−^*. Suggesting that *Ars* has an inhibitory effect on nymph development at specific stages, which is consistent with a previous study in *Drosophila* showing that symbionts can modulate development timelines in insect systems [41,42]. In addition to the developmental delays, we observed significant differences in reproductive performance. In KF30-14 *Ars^+^*, the total population ovipositor (TPOP) was higher than in KF30-14 *Ars^−^*, suggesting that *Ars* may inhibit the reproductive capacity of *N. lugens*. This observation is further supported by lower fecundity, especially in KF30-1*4 Ars^+^* females, and it is consistent with a previous study on spiders infected by *Wolbachia*, which found a significantly shortened lifespan, attributed to metabolic and immune costs. Similarly, our results suggest that symbiont infections can negatively affect host longevity across arthropods [43]. However, reproductive parameters were more favorable in MH86 *Ars^+^*, with higher reproductive values (*v*_xj_) than in KF30-14 *Ars^+^*, highlighting the influence of host plant genotype in modulating symbiont effects.

*Arsenophonus* infection has a significant impact on the expression of genes associated with reproduction and development in *N. lugens*. The RNA sequencing analysis revealed alterations in the expression of genes such as *Tret*, *Vg*, *cyp314a1*, and *JHAMT*, with notable downregulation of *Vg*, *Tret*, and *cyp314a1* in MH86 *Ars*^+^ compared to MH86 *Ars^−^*. Conversely, *JHAMT* was upregulated in KF30-14 *Ars*^+^, indicating that the effect of *Arsenophonus* infection on hormonal pathways may vary between different rice varieties. These findings suggest that *Arsenophonus* infection modulates hormonal and metabolic processes that could reduce fecundity in *N. lugens*, compared with previous studies, including Cai, 2024 [44], which also observed the downregulation of P450 detoxification systems following *Ca. A. Nilaparvata* infection. In their work, *Arsenophonus* was shown to influence insecticide susceptibility by modulating gene expression related to detoxification, thus contributing to increased insecticide sensitivity. Taken together, these studies underscore the role of *Arsenophonus* infection in altering physiological processes of *N. lugens*, potentially influencing both reproduction and pest control strategies. These observations are further supported by the transcriptomic data, particularly DEG and KEGG pathway analysis, which revealed significant enrichment of genes involved in lipid metabolism, amino acid metabolism, and vitamin metabolism in *N. lugens* fed on the transgenic rice variety KF30-14 *Ars***^+^** compared to uninfected KF30-14 *Ars^−^*. These findings provide valuable mechanistic insights into how *Arsenophonus* infection may disrupt essential metabolic pathways, particularly those involved in energy production and nutrient synthesis, which are critical for reproductive success. These results align with the findings of previous studies, which have reported similar alterations in metabolic pathways following symbiont infection in *N. lugens* [45]. In the lipid metabolism pathway, one gene, *long-chain acyl-CoA synthetase*, was found to be differentially expressed, which is a key gene in both fatty acid biosynthesis and degradation. Consistently, similar studies have shown that insect reproduction can affect lipid metabolism, Dong, 2021; Huang, 2023 [46,47], and the hypothesis is that the pathway disruption explains the reduced reproductive capacity in KF30-14 *Ars*^+^ populations. Zhang, 2018 [48] found that interference with *long-chain acyl-CoA synthetase* (*FACL*) eliminated the increased reproductive capacity of *Laodelphax striatellus* induced by *Triazopho*s (*TZP*), suggesting that *FACL* is a key gene in *TZP*-induced reproduction enhancement in brown planthoppers. It is speculated that *long-chain acyl-CoA synthetase* may regulate changes in the egg-laying capacity of brown planthoppers feeding on KF30-14, but its specific effect needs to be validated through interference studies.

Furthermore, the reproductive capacity of KF30-14 *Ars^−^* was higher than that of KF30-14 *Ars*^+^, but the relative expression levels of genes related to *vitellogenin* synthesis, *ecdysteroidogenesis,* and *juvenile* hormone regulation did not differ significantly between the two groups. This implies that *Arsenophonus* does not directly regulate the expression of these genes but rather modulates metabolic pathways like lipid metabolism, which in turn affects the reproductive capacity of *N. lugens*, as reported in Fan, 2016 [49]. Nevertheless, it remains to be determined which specific genes are responsible for these metabolic changes.

## 5. Conclusions

This study highlights the significant role of *Arsenophonus* infection in modulating the development, reproduction, and metabolic processes of *N. lugens*. Our findings demonstrate that *Arsenophonus* infection leads to delayed development, reduced fecundity, and alterations in reproductive organ size, particularly in insects fed on the transgenic rice variety. These results are supported by quantitative data from life table analysis, where infected populations exhibited lower reproductive capacity and slower developmental rates compared to uninfected counterparts. Furthermore, RNA sequencing revealed that *Arsenophonus* infection significantly impacted the expression of genes involved in lipid metabolism, which may explain the observed effects on reproduction. The data presented in this study provide valuable insights into the complex interactions between symbiotic bacteria and their insect hosts, particularly in the context of genetically modified crops. Future pest management strategies could leverage these interactions, using symbiotic bacteria to influence pest populations in a more environmentally friendly manner. Modulating microbial communities could serve as a novel approach for controlling agricultural pests and reducing reliance on chemical pesticides.

## Figures and Tables

**Table 1:** Developmental Stages, Longevity, and Statistical Analysis of *N. lugens* Populations fed on MH86 and KF30-14 rice varieties with *Arsenophonus* infection.

Development Stage	Rice Line	*Arsenophonus* ^−^		*Arsenophonus* ^+^	
		*n*	Duration/d	*n*	Duration/d
1st instar nymph	MH86	80	2.96 ± 0.06 *	80	3.41 ± 0.08 a
	KF30-14	82	2.98 ± 0.05	76	3.13 ± 0.06 b
2nd instar nymph	MH86	74	2.46 ± 0.09	76	2.45 ± 0.09
	KF30-14	77	2.29 ± 0.07	62	2.50 ± 0.08
3rd instar nymph	MH86	69	2.54 ± 0.09	76	2.41 ± 0.07 b
	KF30-14	73	2.49 ± 0.07 *	54	2.74 ± 0.10 a
4th instar nymph	MH86	62	2.87 ± 0.10 a	75	2.71 ± 0.09
	KF30-14	69	2.61 ± 0.07 b*	50	2.88 ± 0.10
5th instar nymph	MH86	56	4.25 ± 0.20	69	4.16 ± 0.13 b
	KF30-14	65	4.34 ± 0.08	44	4.89 ± 0.27 a
Adult longevity	MH86	56	19.46 ± 1.44	69	21.02 ± 1.15
	KF30-14	65	22.84 ± 1.16	44	22.70 ± 1.41
Male adult longevity	MH86	21	22.10 ± 2.84	28	25.36 ± 1.91
	KF30-14	28	21.61 ± 2.03	17	23.35 ± 2.53
Female adult longevity	MH86	35	17.89 ± 1.52 b	41	18.07 ± 1.25 b
	KF30-14	37	23.78 ± 1.35 a	27	22.30 ± 1.69 a
Total longevity	MH86	89	23.97 ± 1.71 b*	84	30.93 ± 1.55 a
	KF30-14	84	30.57 ± 1.68 a	83	23.34 ± 1.97 b*

Data in the table are mean ± SE; *n* represents the sample size. * Indicated that *Nilaparvata lugens* fed on the same rice variety, and there was a significant difference between treatments with and without carrying *Arsenophonus* (*p* < 0.05). Different lowercase letters indicated that the *Arsenophonus* treatment was the same, and there was a significant difference among the treatments fed on different rice varieties (*p* < 0.05).

**Table 2:** Reproductive parameters of *N. lugens* populations fed on MH86 and KF30-14 rice varieties with *Arsenophonus* infection.

Parameters	Rice Line		*Arsenophonus*^−^ Duration/d		*Arsenophonus^+^* Duration/d
		n		n	
Adult pre-oviposition period (APOP)	MH86	33	2.97 ± 0.27 a	39	2.67 ± 0.16 b
	KF30-14	37	3.51 ± 0.23 a*	27	4.33 ± 0.30 a
Total preoviposition period (TPOP)	MH86	33	18.36 ± 0.63 a	39	18.03 ± 0.31 b
	KF30-14	37	18.41 ± 0.28 a*	27	20.41 ± 0.51 a
Oviposition days (Od)	MH86	33	13.33 ± 46 a	39	14.18 ± 1.33 a
	KF30-14	37	17.16 ± 1.40 a	27	14.22 ± 1.40 a
Fecundity	MH86	33	349.12 ± 43.87 a	39	407.72 ± 44.86 a
	KF30-14	37	373.62 ± 39.04 a	27	255.93 ± 34.78 b*

Data in the table are mean ± SE; *n* represents the sample size. * Indicated that *Nilaparvata lugens* fed on the same rice variety, and there was a significant difference between treatments with and without carrying *Arsenophonus* (*p* < 0.05). Different lowercase letters indicated that the *Arsenophonus* treatment was the same, and there was a significant difference among the treatments fed on different rice varieties (*p* < 0.05).

**Table 3:** The mortality distribution in percentage of *Nilaparvata lugens.* Populations fed on MH86 and KF30-14 rice varieties with *Arsenophonus* infection.

Development Stage	Rice Line	*Arsenophonus* ^−^	*Arsenophonus^+^*
1st instar nymph	MH86	0.10 ± 0.03 a	0.05 ± 0.02 a
	KF30-14	0.02 ± 0.02 b	0.08 ± 0.03 a
2nd instar nymph	MH86	0.07 ± 0.03 a	0.05 ± 0.02 b
	KF30-14	0.06 ± 0.03 a	0.17 ± 0.04 a
3rd instar nymph	MH86	0.06 ± 0.02 a	0.00 ± 0.00 b*
	KF30-14	0.05 ± 0.02 a	0.10 ± 0.03 a
4th instar nymph	MH86	0.08 ± 0.03 a	0.01 ± 0.01 a*
	KF30-14	0.05 ± 0.02 a	0.05 ± 0.02 a
5th instar nymph	MH86	0.07 ± 0.03 a	0.07 ± 0.03 a
	KF30-14	0.05 ± 0.02 a	0.07 ± 0.03 a
Immature	MH86	0.37 ± 0.05 a	0.18 ± 0.04 b*
	KF30-14	0.23 ± 0.05 b*	0.47 ± 0.05 a
Female adult	MH86	0.39 ± 0.05 a	0.49 ± 0.05 a
	KF30-14	0.44 ± 0.05 a	0.33 ± 0.05 b
Male adult	MH86	0.24 ± 0.04 a	0.33 ± 0.05 a
	KF30-14	0.33 ± 0.05 a	0.20 ± 0.04 a
Adult	MH86	0.63 ± 0.05 b*	0.82 ± 0.04 a
	KF30-14	0.77 ± 0.05 a	0.53 ± 0.05 b*

Data in the table are mean ± SE. * Indicated that *Nilaparvata lugens* fed on the same rice variety, and there was a significant difference between treatments with and without carrying *Arsenophonus* (*p* < 0.05). Different lowercase letters indicated that the *Arsenophonus* treatment was the same, and there was a significant difference among the treatments fed on different rice varieties (*p* < 0.05).

**Table 4:** Population parameters of *Nilaparvata lugens* under different treatments.

Population Parameters	Rice Varieties	*Arsenophonus* ^−^	*Arsenophonus^+^*
Intrinsic rate of increase (d^−1^)	MH86	0.204 ± 0.009 a	0.218 ± 0.008 a
	KF30-14	0.202 ± 0.007 a	0.167 ± 0.010 b*
Mean generation time (d^−1^)	MH86	23.805 ± 0.432 b	24.041 ± 0.311 b
	KF30-14	25.272 ± 0.410 a	26.340 ± 0.720 a
Net reproductive rate	MH86	129.449 ± 24.021 a	189.298 ± 30.262 a
	KF30-14	164.571 ± 26.67 a	83.253 ± 18.237 b*

Data in the table are mean ± SE. * Indicated that *Nilaparvata lugens* fed on the same rice variety, and there was a significant difference between treatments with and without carrying *Arsenophonus* (*p* < 0.05). Different lowercase letters indicated that the *Arsenophonus* treatment was the same, and there was a significant difference among the treatments fed on different rice varieties (*p* < 0.05).

## Data Availability

The data sets used or analyzed during current study are available from the corresponding author upon reasonable request.

## Supplementary Materials

The following supporting information can be downloaded at: https://www.mdpi.com/article/10.3390/insects17020222/s1, Figure S1: PCR confirmation of *Arsenophonus* infection in eggs of *Nilaparvata lugens*. Lane M shows the DL2000 DNA marker. Lanes 1–9 contain egg samples collected from *N. lugens* fed on KF30-14; lanes 1, 2, 8, and 9 show no detectable amplification and are considered *Arsenophonus*-negative. Lanes 10–17 contain egg samples collected from *N. lugens* fed on MH86; Lanes 10, 11, 12, 14, and 16 are negative. The presence of a distinct PCR band indicates *Arsenophonus* infection in the corresponding egg samples; Figure S2: Schematic representation of the procedure used to establish *Arsenophonus*-infected (*Ars*⁺) and uninfected (*Ars*⁻) colonies of *Nilaparvata lugens* fed on two rice varieties (MH86 and KF30-14). Eggs from PCR-confirmed females were maintained in test tubes on rice seedlings to establish the main colonies. DNA was extracted from female adults, and *Arsenophonus* infection was verified by PCR amplification and sequencing. Based on PCR results, eggs from *Arsenophonus*-positive females were used to maintain the *Ars*⁺ line, while eggs from negative females were used to establish the *Ars*⁻ line. Rice seedlings reared in test tubes were periodically transferred to new cages for colony maintenance; Figure S3: Phylogenetic tree of *Arsenophonus* strains in *Nilaparvata lugens* and related insect species. (★) the gene sequence of *Arsenophonus* in *Nilaparvata lugens* fed on KF30-14; (▲) the gene sequence of *Arsenophonus* in *Nilaparvata lugens* fed on MH86; Figure S4: Distribution of transcript lengths after assembly (Map of sequence length in transcript after transcriptome sequence assembly); Figure S5: Gene annotation results from various databases “The results of transcriptome assembly were compared with six databases (GO, KEGG, EggNOG, NR, Swiss-Prot, and Pfam), yielding 12,103—9818—14,596—18,089—11,796, and 13,417 genes, respectively. Among these, the NR database had the highest number of matched genes”; Figure S6: Number of differentially expressed genes in each comparison group; Figure S7: Volcano plots of DEGs in each comparison group. (A) the volcano plot of DEGs in MH86 *Ars^−^* vs MH86 *Ars*^+^; (B) the volcano plot of DEGs in KF30-14 *Ars^−^* vs KF30-14 *Ars*^+^; (C) the volcano plot of DEGs in MH86 *Ars^−^*vs KF30-14 *Ars^−^*; (D) the volcano plot of DEGs in MH86 *Ars*^-^ vs KF30-14 *Ars*^+^; (E) the volcano plot of DEGs in MH86 *Ars*^+^ vs KF30-14 *Ars^−^*; (F) the volcano plot of DEGs in MH86 *Ars*^+^ vs KF30-14 *Ars*^+^; Figure S8: EggNOG Annotated classified statistical charts. The same description with table 4, 5; Figure S9: GO annotation analysis of DEGs; Fig S10: KEGG annotation analysis. (A) the annotation analysis of DEGs in MH86 *Ars*-vs MH86 *Ars*^+^; (B) the annotation analysis of DEGs in KF30-14 *Ars*^-^ vs KF30-14 *Ars*^+^; (C) the annotation analysis of DEGs in MH86 *Ars^−^* vs KF30-14 *Ars*^-^; (D) the annotation analysis of DEGs in MH86 *Ars*^+^ vs KF30-14 *Ars*^+^; (E) the annotation analysis of DEGs in MH86 *Ars^−^* vs KF30-14 *Ars*^+^; (F) the annotation analysis of DEGs in MH86 *Ars*^+^ vs KF30-14 *Ars*^-^; Figure S11: Venn diagram of differentially expressed genes between treatment groups; Figure S12: KEGG enrichment analysis of DEGs in each treatment comparison. (A) the enrichment results of DEGs in MH86 *Ars^−^* vs MH86 *Ars*^+^; (B) the enrichment results of DEGs in KF30-14 *Ars^−^* vs KF30-14 *Ars*^+^; (C) the enrichment results of DEGs in MH86 *Ars*^-^ vs KF30-14 *Ars^−^* ; (D) the enrichment results of DEGs in MH86 *Ars*^+^ vs KF30-14 *Ars*^+^; (E) the enrichment results of DEGs in MH86 *Ars^−^* vs KF30-14 *Ars*^+^; (F) the enrichment results of DEGs in MH86 *Ars*^+^ vs KF30-14 *Ars^−^*; Table S1 Primers sequence; Table S2 Computational formula; Table S3 qRT-PCR primers; Table S4: RNA quality test results for samples from different treatments; Table S5: Transcriptome data quality control results for sequencing; Table S6: Mapping ratio statistics for transcriptome alignment.
