# Supplementary material for: Developmental and Reproductive Impacts of Arsenophonus Symbiont on the Population of Nilaparvata lugens
Source: Insects. 2026 Feb 20;17(2):222. doi: 10.3390/insects17020222 (PMC12942160; doi:10.3390/insects17020222)
Supplement: Supplementary file 1 [file insects-17-00222-s001.zip › insects-4116994-supplementary.pdf]

Supplementary Material

Establishment of *N. lugens* Infected Populations and Bacterial Detection

In this study, the *Arsenophonus*-negative lines were experimentally established. We used antibiotic treatment to eliminate *Arsenophonus* from the insect populations. The process involved exposing *N. lugens* to rice seedlings treated with 25 μg/mL ampicillin for five consecutive generations. This antibiotic was chosen based on its effectiveness in disrupting the gut microbiota of the insects, which was confirmed through previous studies [24] [25] [26] . The experiment combined two rice varieties (MH86 and KF30-14). The rice varieties were used to differentiate between transgenic Bt rice (KF30-14) and the non-transgenic control (MH86), while ampicillin exposure was employed to eliminate *Arsenophonus*. To ensure the antibiotic's effectiveness in eliminating the symbiont, adult insects from each rice variety were reared on ampicillin-treated rice seedlings for five generations. The protocol for antibiotic treatment included exposing rice seedlings to the antibiotic, followed by transferring the fifth-instar nymphs onto these treated seedlings. The insects were maintained on antibiotic-treated plants throughout their development, ensuring consistent disruption of their gut microbiota over multiple generations. After five generations, to distinguish between *Arsenophonus*-positive and *Arsenophonus*-negative individuals. Fifth-instar nymphs were selected from laboratory colonies that had been maintained on the MH86 and KF30-14 rice. Individual nymphs were placed singly into glass test tubes (15 cm × 2.5 cm) and reared to adulthood. Emerged adults were paired (one male + one female) in new tubes; one week after pairing females were collected and their DNA extracted for *Arsenophonus* detection. DNA was extracted using the Fast Pure Cell/Tissue DNA Isolation Mini Kit (Nanjing Novogene Bio-tech Co., Ltd.) following the manufacturer's protocol (tissue disruption, enzymatic lysis, binding, and wash and elution steps as described in the kit instructions). Extracted DNA was used as template for PCR detection with *Arsenophonus*-specific primers (Table S1), using Phanta Max high-fidelity polymerase. The PCR reaction mix and cycling conditions were: 12.5 μL 2× Phanta Max Buffer, 0.5 μL dNTP Mix (10 mM), 1.0 μL forward primer, 1.0 μL reverse primer, 0.5 μL Phanta® Max polymerase, 2.0 μL DNA, and nuclease-free water to 25 μL; PCR program: 95 °C 3 min; 35 cycles of 95 °C 30 s, 60 °C 30 s, 72 °C 1 min; final extension 72 °C 10 min. PCR products were checked by agarose gel electrophoresis, positive bands were purified, sequenced and confirmed by BLAST comparison in NCBI. The *Arsenophonus* infection status of females and their eggs was determined and confirmed by PCR analysis. Based on these results, *Arsenophonus*-positive (*Ars*$^{+}$) and *Arsenophonus*-negative (*Ars*$^{-}$) lines were established and maintained on their respective rice varieties. (Figure 1 and Figure S1). Offspring (F1 generation) from 7*N. lugens Ars*$^{+}$, and 7 *N. lugens Ars*$^{-}$, have used to establish the colonies as experimental materials. All lines were maintained in an artificial climate chamber at 26 ± 1 °C, photoperiod 16L: 8D, and relative humidity 80% ± 1% for subsequent experiments. Additionally, reciprocal crossing experiments between positive and negative adults demonstrated that infection was inherited exclusively through the maternal line, confirming that *Arsenophonus* is vertically transmitted via the eggs. Consequently, the infected and uninfected lines used in this study were maintained through maternal inheritance, with infection status in each generation verified by diagnostic PCR amplification of 23S rDNA fragment specific to *Arsenophonus* as explained in the simple diagram Figure S1 and S2.

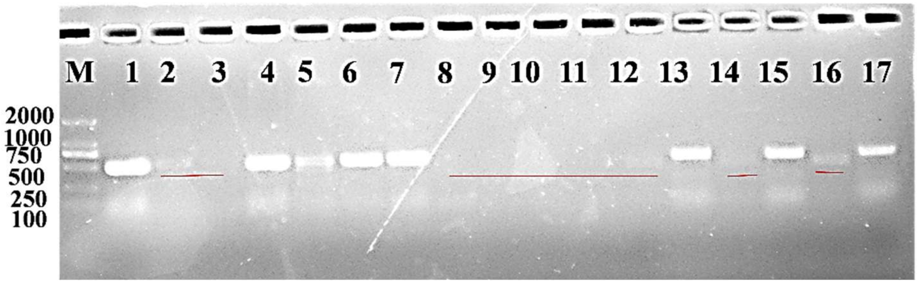

Figure S1: PCR confirmation of *Arsenophonus* infection in eggs of *Nilaparvata lugens*. Lane M shows the DL2000 DNA marker. Lanes 1–9 contain egg samples collected from *N. lugens* fed on KF30-14; lanes 1, 2, 8, and 9 show no detectable amplification and are considered *Arsenophonus*-negative. Lanes 10–17 contain egg samples collected from *N. lugens* fed on MH86; Lanes 10, 11, 12, 14, and 16 are negative. The presence of a distinct PCR band indicates *Arsenophonus* infection in the corresponding egg samples.

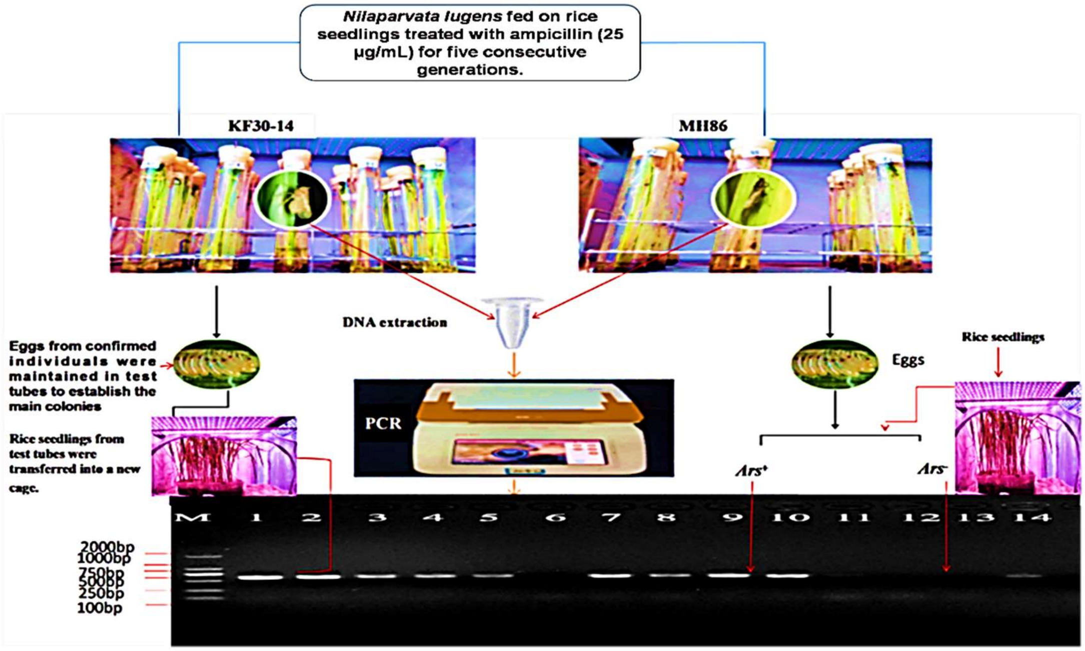

Figure S2: Schematic representation of the procedure used to establish *Arsenophonus*-infected (*Ars*$^+$) and uninfected (*Ars*$^-$) colonies of *Nilaparvata lugens* fed on two rice varieties (MH86 and KF30-14). Eggs from PCR-confirmed females were maintained in test tubes on rice seedlings to establish the main colonies. DNA was extracted from female adults, and *Arsenophonus* infection was verified by PCR amplification and sequencing. Based on PCR results, eggs from *Arsenophonus*-positive females were used to maintain the *Ars*$^+$ line, while eggs from negative females were used to establish the *Ars*$^-$ line. Rice seedlings reared in test tubes were periodically transferred to new cages for colony maintenance.

Table S1 Primers sequence

Primer name	Primer sequence (5′-3′)
**ARS23S-F**	CGTTTGATGAATTCATAGTCAAA
**ARS25S-R**	GGTCCTCCAGTTAGTGTTACCCAAC

Phylogenetic Analysis of *Arsenophonus* in *Nilaparvata lugens*

We compared the gene sequences of *Arsenophonus* detected in the MH86 and KF30-14 *Nilaparvata lugens* to construct a phylogenetic tree. The results indicated that the *Arsenophonus* strains from both *Nilaparvata lugens* populations (MH86 and KF30-14) were closely related, belonging to the same strain. Phylogenetic analysis further showed that these *Arsenophonus* strains were most closely related to those found in other insect species, particularly *Arsenophonus symbionts* from the whitefly family (*Aleyrodidae*), specifically *Tetraleurodes acaciae*, *Acanthaleyrodesstyraci*, and from the planthoppers family (*Delphacidae*), particularly *Nilaparvata lugens*. In contrast, *Arsenophonus* strains in *Bemisia tabaci* (whitefly species) were more distantly related as shown in (**Figure S3)**.

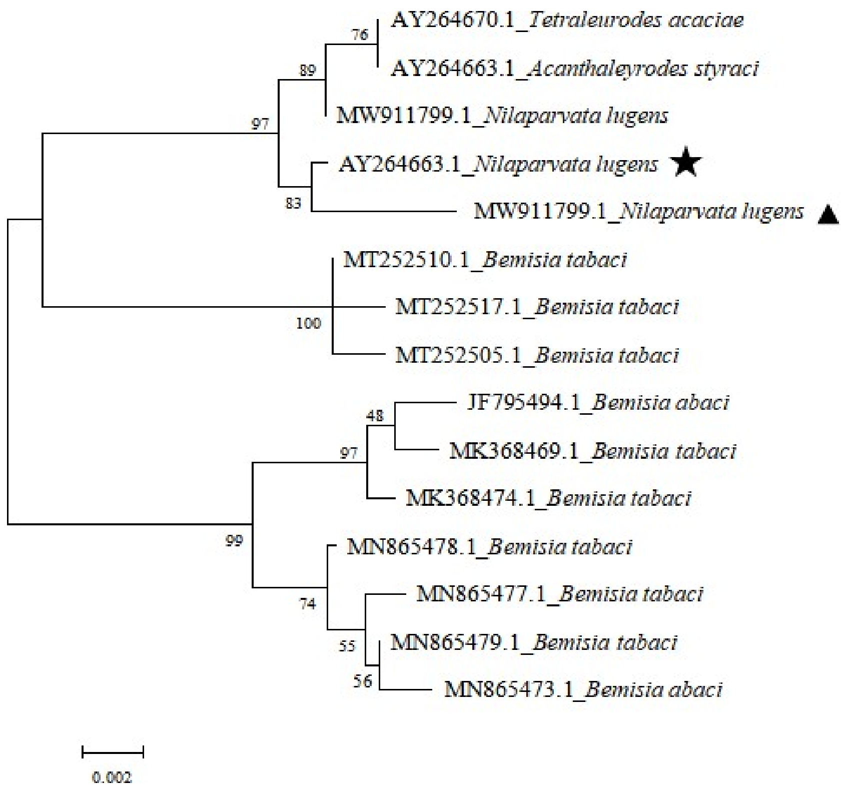

Figure S3: Phylogenetic tree of *Arsenophonus* strains in *Nilaparvata lugens* and related insect species. (★) the gene sequence of *Arsenophonus* in *Nilaparvata lugens* fed on KF30-14; (▲) the gene sequence of *Arsenophonus* in *Nilaparvata lugens* fed on MH86.

Construction of the Two-Sex Life Table for the *Nilaparvata lugens* Population

Measurement of the fitness of *Nilaparvata lugens* populations

Population fitness is a concept related to a group and refers to the ability of a biological population to survive and pass on its acquired characteristics to the next generation in a specific ecological environment. This ability generally includes vitality and reproductive capacity. Vitality is usually expressed by survival rate, lifespan, and growth development speed, while reproductive capacity is typically indicated by the number of offspring produced. Based on the theory of the age-stage two-sex life table, we constructed age-

stage two-sex life tables for different treatments of *N. lugens.* Five pairs of *N. lugens* from each population were placed in test tubes for rearing. After one week, the hatching of eggs in the test tubes was observed daily. Once nymphs were observed hatching, newly hatched nymphs from each treatment were individually transferred to corresponding rice varieties for rearing, with 90 individuals per treatment. Daily observations and recordings of molting, mortality, and emergence of *N. lugens* were made. Within 12 hours of emergence, *N. lugens* was paired. After pairing, fresh rice seedlings were provided daily, and the replaced seedlings were examined under a microscope to count and record the number of eggs until all test brown planthoppers died.

Data Statistics and Analysis…

Based on the constructed age-stage two-sex life table, calculate the age-stage specific survival rate, female adult age-stage specific fecundity (f_{xj}), age-specific survival rate (l_x), age-specific fecundity (m_x), and age-stage. Expected longevity (e_{xj}), intrinsic growth rate (γ), reproductive value (v_{xj}), finite rate of increase (λ), net reproductive rate (R_0), mean generation time (T), and population prediction. The specific calculation formulas are shown in **Table S2.**

Table S2 Computational formula

Parameter	Formula
l_x is the survival rate of the population from egg to age x."	$l_x = \sum_{j=1}^{m} s_{xj}$
m_x is the average egg production of the population at age x."	$m_x = \frac{\sum_{j=1}^{m} s_{xj} f_{xj}}{\sum_{j=1}^{m} s_{xj}}$
e_{xj} is the total expected number of days an individual in the population will survive from age x to stage j.	$e_{xj} = \sum_{i=x}^{\infty} \sum_{y=j}^{m} s'_{iy}$
v_{xj} is the contribution of individuals at age x and stage j to the future population.	$v_{xj} = \frac{e^{r(x+1)}}{s_{xj}} \sum_{i=x}^{\infty} e^{-r(i+1)} \sum_{y=j}^{m} s'_{iy} f_{iy}$
γ is the maximum growth capacity of the population under favorable environmental conditions, with sufficient food, and the exclusion of unfavorable factors.	$\sum_{x=0}^{\infty} e^{-r(x+1)} l_x m_x = 1$
R_0, net reproductive rate, is the total number of offspring produced by an individual throughout its lifetime."	$R_0 = \sum_{x=0}^{\infty} l_x m_x$

T, the time required for the population to increase by a factor of R0 when it reaches a stable age-stage distribution and stable growth rate	$T = \frac{\ln R_0}{r}$
Population prediction	$\phi_{j,t} = \frac{\log(n_{j,t+1} + 1)}{\log(n_{j,t} + 1)}$ $r_{j,t} = \ln\left(\frac{n_{j,t+1} + 1}{n_{j,t} + 1}\right) = \ln(n_{j,t+1} + 1) - \ln(n_{j,t} + 1)$

The basic parameters of the life table data were calculated using the TWOSEX-MSChart software program (Chi, 2022). The Bootstrap method was used to assess the mean and standard error, with 100,000 bootstrap replications. The Paired Bootstrap Test in the software was used to test the differences between the data. The Timing program was used to predict the population dynamics of the brown planthopper over the next 60 days. Sigma Plot 14.0 was used for graphing.

Table S3 qRT-PCR primers

Primer name	Primer sequence (5′-3′)	Gene accession
q-*Vg*-F	CACTGATCTACTTCTCGCTG	XM_039439358.1
q-*Vg*-R	CTCATCCCCCTCGTAGATAT	
q-*JHAMT*-F	ATGCAGCGGGATATGATTTG	XM_039428318.1
q-*JHAMT*-R	CTGACTGCTATTTCGATTCTCGT	
q-*cyp314a1*-F	TGTCAGTTGGATCGTGCAG	XM_039433591.1
q-*cyp314a1*-R	ACAGGATATCGCTGGCTT	
q-*Tret*-F	CCTGCTGGGTGTGATTGGAT	XM-022329823.1
q-*Tret*-R	CCATACCCTTGACGTCCTCG	
q-*actin*-F	TGGACTTCGAGCAGGAAATGG	XM_022345417.2
q-*actin*-R	ACGTCGCACTTCATGATC	

Transcriptome Analysis

RNA Quality Detection and Sequencing

RNA was successfully extracted from female adult *Nilaparvata lugens* samples across four treatment groups: MH86 *Ars*$^{+}$, MH86 *Ars*$^{-}$, KF30-14 *Ars*$^{+}$, and KF30-14 *Ars*$^{-}$. A total of 12 samples were collected after the first molting. RNA quality assessments showed that the concentrations and total amounts met the requirements for transcriptome sequencing (Table S4). Transcriptome sequencing generated a total of 85.44 GB of clean data across the 12 samples, with each sample yielding over 6.36 GB. The sequencing data quality was high, with Q20 percentages exceeding 98%, Q30 percentages exceeding 95%, and error rates below 0.015, meeting the standards for further biological analysis (Table S5).

Table S4: RNA quality test results for samples from different treatments.

Sample name	Concentration （ng/μL）	Total amount （μg）	OD$_{260/280}$	OD$_{260/230}$
**MH86 *Ars*$^{-}$1**	974.1	34.09	1.83	2.39
**MH86 *Ars*$^{-}$2**	1116.58	39.08	1.85	2.39
**MH86 *Ars*$^{-}$3**	1067.18	37.35	1.98	2.35
**MH86 *Ars*$^{+}$4**	955.27	33.43	1.83	2.39
**MH86 *Ars*$^{+}$5**	1042.35	88.6	1.9	2.47
**MH86 *Ars*$^{+}$6**	1207.02	102.6	1.9	2.45
**KF30-14 *Ars*$^{-}$1**	1210.91	102.93	1.92	2.42
**KF30-14 *Ars*$^{-}$2**	736.59	25.78	1.81	2.39
**KF30-14 *Ars*$^{-}$3**	1267.71	44.37	1.92	2.33
**KF30-14 *Ars*+5**	1357.71	47.52	1.86	2.34
**KF30-14 *Ars*$^{+}$6**	1373.14	48.06	1.86	2.35
**KF30-14 *Ars*$^{+}$7**	1358.04	7.53	1.85	2.36

MH86 *Ars*$^{-}$, *Nilaparvata lugens* without *Arsenophonus* fed on MH86; MH86 *Ars*$^{+,}$ *Nilaparvata lugens* with *Arsenophonus* fed on MH86; KF30-14 *Ars*$^{-}$, *Nilaparvata lugens* without *Arsenophonus* fed on KF30-14; KF30-14 *Ars*$^{+}$, *Nilaparvata lugens* with *Arsenophonus* fed on KF30-14.

Table S5: Transcriptome data quality control results for sequencing.

Sample	Raw reads	Clean reads	Error rate (%)	Q20 (%)	Q30 (%)
MH86 *Ars*$^{-}$ 1	45315644	44983564	0.0125	98.44	95.1
MH86 *Ars*$^{-}$ 2	44675840	44350086	0.0125	98.44	95.12
MH86 *Ars*$^{-}$ 3	45266688	44928822	0.0124	98.48	95.25

MH86 *Ars*⁺ 1	45920460	45606578	0.0124	98.47	95.23
MH86 *Ars*⁺ 2	50686090	50377120	0.0124	98.5	95.3
MH86 *Ars*⁺ 3	42831190	42539512	0.0125	98.42	95.04
KF30-14 *Ars*⁻ 1	53185458	52808046	0.0125	98.47	95.21
KF30-14 *Ars*⁻ 2	52523248	52179732	0.0124	98.51	95.29
KF30-14 *Ars*⁻ 3	47269124	46933232	0.0125	98.46	95.18
KF30-14 *Ars*⁺ 1	49218642	48855136	0.0125	98.45	95.17
KF30-14 *Ars*⁺ 2	47786492	47481938	0.0123	98.55	95.42
KF30-14 *Ars*⁺ 3	50174998	49817128	0.0124	98.5	95.3

Sequence Alignment and Assembly

The clean data were aligned to the reference genome, achieving mapping rates between 80.23% and 82.69%, confirming the appropriateness of the reference genome and the absence of contamination in the sequencing process (Table 6). After quality control, 55,748 transcripts were assembled, of which 37,544 matched known transcripts from the reference annotation. The majority of the transcripts had a length greater than 1800 bp (30,619 transcripts), followed by those with lengths between 201-400 bp (3,592 transcripts), and the fewest were between 1-200 bp (735 transcripts) (**Figure** S4).

Table S6: Mapping ratio statistics for transcriptome alignment.

Sample	Total reads	Total mapped (%)	Multiple mapped (%)	Uniquely mapped (%)
**MH86 *Ars*⁻ 1**	44983564	36595042(81.35)	8132189(18.08)	28462853(63.27)
**MH86 *Ars*⁻ 2**	44350086	35584156(80.23)	8265371(18.64)	27318785(61.6)
**MH86 *Ars*⁻ 3**	44928822	36369268(80.95)	8012362(17.83)	28356906(63.12)
**MH86 *Ars*⁺ 1**	45606578	37710118(82.69)	9516253(20.87)	28193865(61.82)
**MH86 *Ars*⁺ 2**	50377120	40725688(80.84)	8263782(16.4)	32461906(64.44)
**MH86 *Ars*⁺ 3**	42539512	34437578(80.95)	6806132(16.0)	27631446(64.95)
**KF30-14 *Ars*⁻ 1**	52808046	43473940(82.32)	9987889(18.91)	33486051(63.41)
**KF30-14 *Ars*⁻ 2**	52179732	42972039(82.35)	10133275(19.42)	32838764(62.93)

**KF30-14 *Ars*⁻ 3**	46933232	38210117(81.41)	7831618(16.69)	30378499(64.73)
**KF30-14 *Ars*⁺ 1**	48855136	39760516(81.38)	8276376(16.94)	31484140(64.44)
**KF30-14 *Ars*⁺ 2**	47481938	38926622(81.98)	8633261(18.18)	30293361(63.8)
**KF30-14 *Ars*⁺ 3**	49817128	40758583(81.82%)	8935547(17.94)	31823036(63.88)

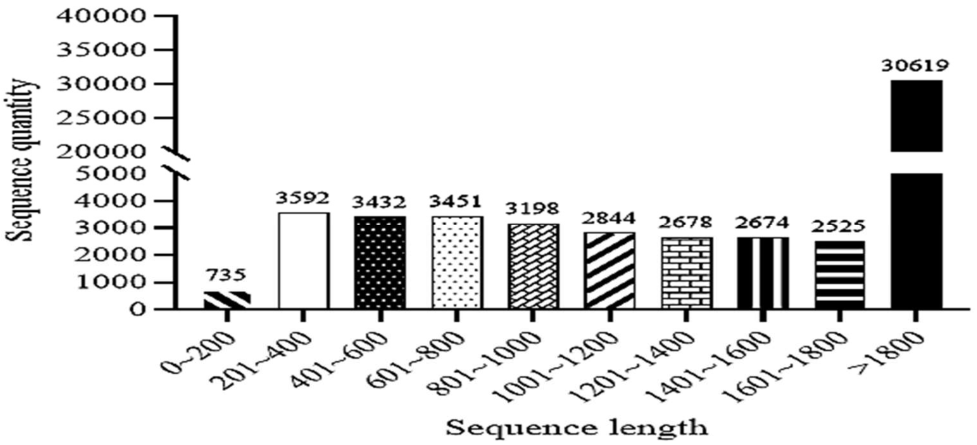

Figure S4: Distribution of transcript lengths after assembly (Map of sequence length in transcript after transcriptome sequence assembly).

Gene Annotation and Number of DEGs

The assembled transcriptome data were annotated using six comprehensive databases: Gene Ontology (GO), Kyoto Encyclopedia of Genes and Genomes (KEGG), EggNOG, NR, Swiss-Prot, and Pfam. Among these, the NR database yielding the highest number of matches (18,089 genes), indicating robust genome mapping and functional annotation (**Figures S5**). Differential expression analysis across treatment groups revealed significant transcriptional changes induced by *Arsenophonus* infection and host plant variation (**Figures S6 and S7A–F**). A total of 462 DEGs were identified in MH86 *Ars*$^-$ vs MH86 *Ars*$^+$, 609 in KF30-14 *Ars*$^-$ vs KF30-14 *Ars*$^+$, 395 in MH86 *Ars*$^-$ vs KF30-14 *Ars*$^-$, 560 in MH86 *Ars*$^-$ vs KF30-14 *Ars*$^+$, 638 in MH86 *Ars*$^+$ vs KF30-14 *Ars*$^-$, and 495 in MH86 *Ars*$^+$ vs KF30-14 *Ars*$^+$. These findings highlight key pathways involved in lipid metabolism, amino acid metabolism, and other biological processes relevant to the physiological changes observed in *N. lugens*.

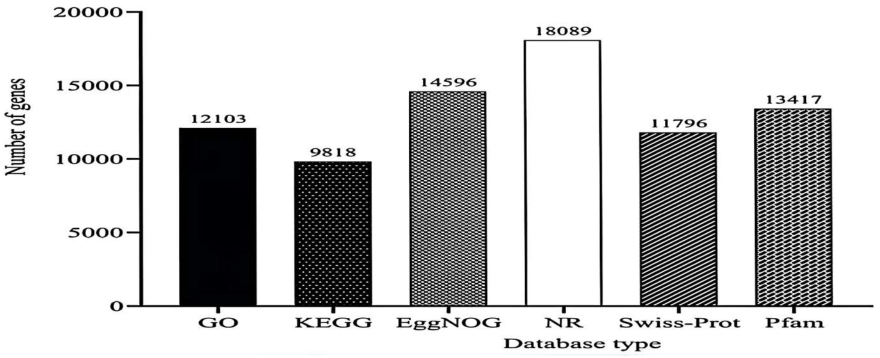

Figure S5: Gene annotation results from various databases "The results of transcriptome assembly were compared with six databases (**GO, KEGG, EggNOG, NR, Swiss-Prot, and Pfam**), yielding **12,103 - 9,818 - 14,596 -18,089 - 11,796, and 13,417 genes**, respectively. Among these, the **NR database** had the highest number of matched genes".

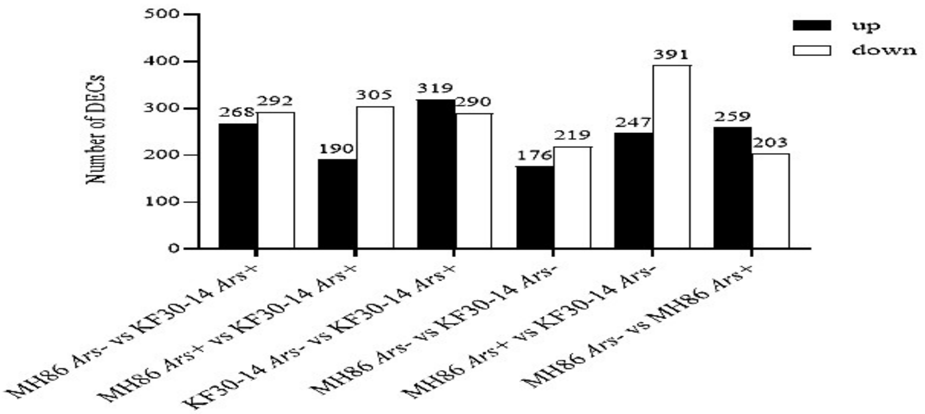

Figure S6: Number of differentially expressed genes in each comparison group.

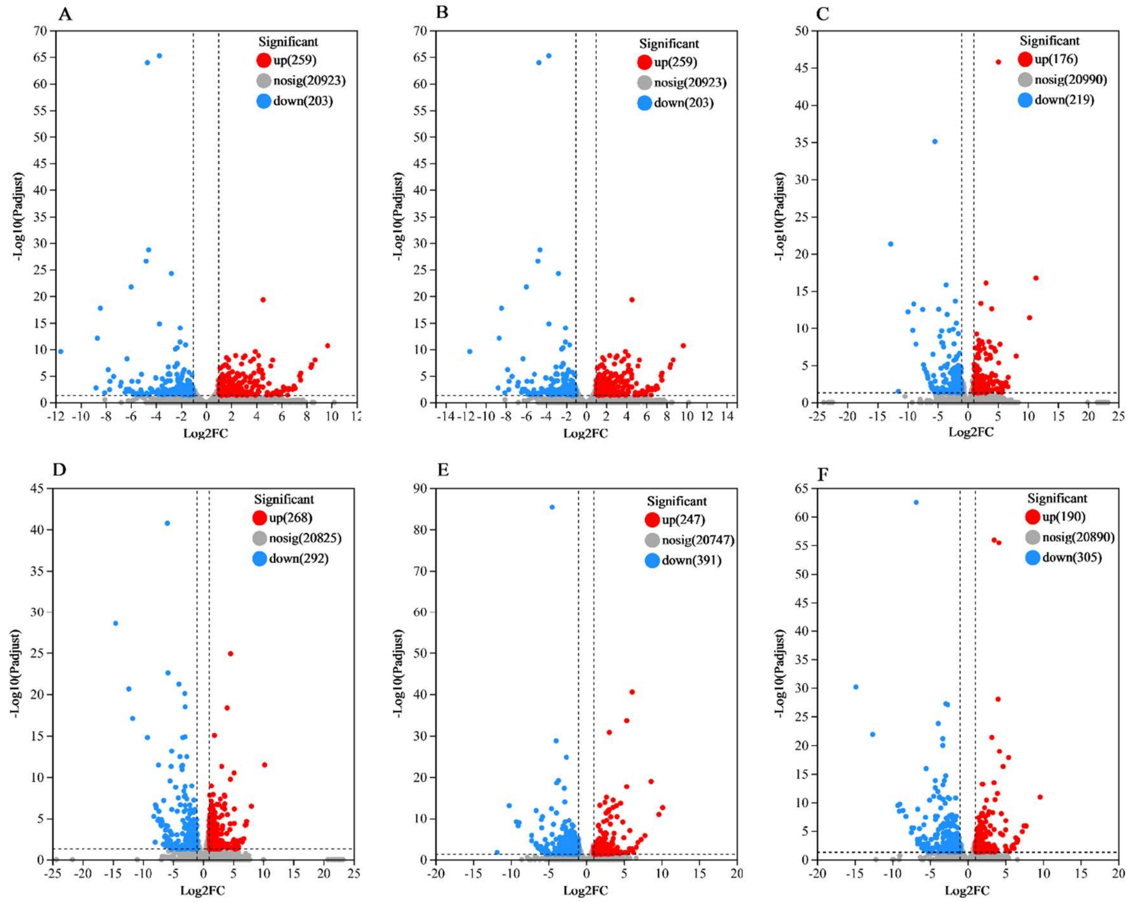

Figure S7: Volcano plots of DEGs in each comparison group. (A) the volcano plot of DEGs in MH86 *Ars*$^-$ vs MH86 *Ars*$^+$; (B) the volcano plot of DEGs in KF30-14 *Ars*$^-$ vs KF30-14 *Ars*$^+$; (C) the volcano plot of DEGs in MH86 *Ars*$^-$ vs KF30-14 *Ars*$^-$; (D) the volcano plot of DEGs in MH86 *Ars*$^-$ vs KF30-14 *Ars*$^+$; (E) the volcano plot of DEGs in MH86 *Ars*$^+$ vs KF30-14 *Ars*$^-$; (F) the volcano plot of DEGs in MH86 *Ars*$^+$ vs KF30-14 *Ars*$^+$.

Gene Functional Annotation Analysis

The EggNOG annotation analysis identified the functional categories of DEGs in the pair wise comparisons. The most frequent categories included protein post-translational modification, protein turnover, and chaperone functions. Genes involved in replication, recombination, and repair processes were the second most frequent category (**Figure S8**). GO annotation analysis revealed that the genes were primarily involved in three main functional categories: biological processes, cellular differentiation, and molecular functions. Within biological processes, genes were most associated with cellular processes, metabolic processes, and biological regulation. In cellular differentiation, genes were linked to membrane parts and cell components. Molecular functions were primarily related to catalytic activity and binding (**Figure S9**).

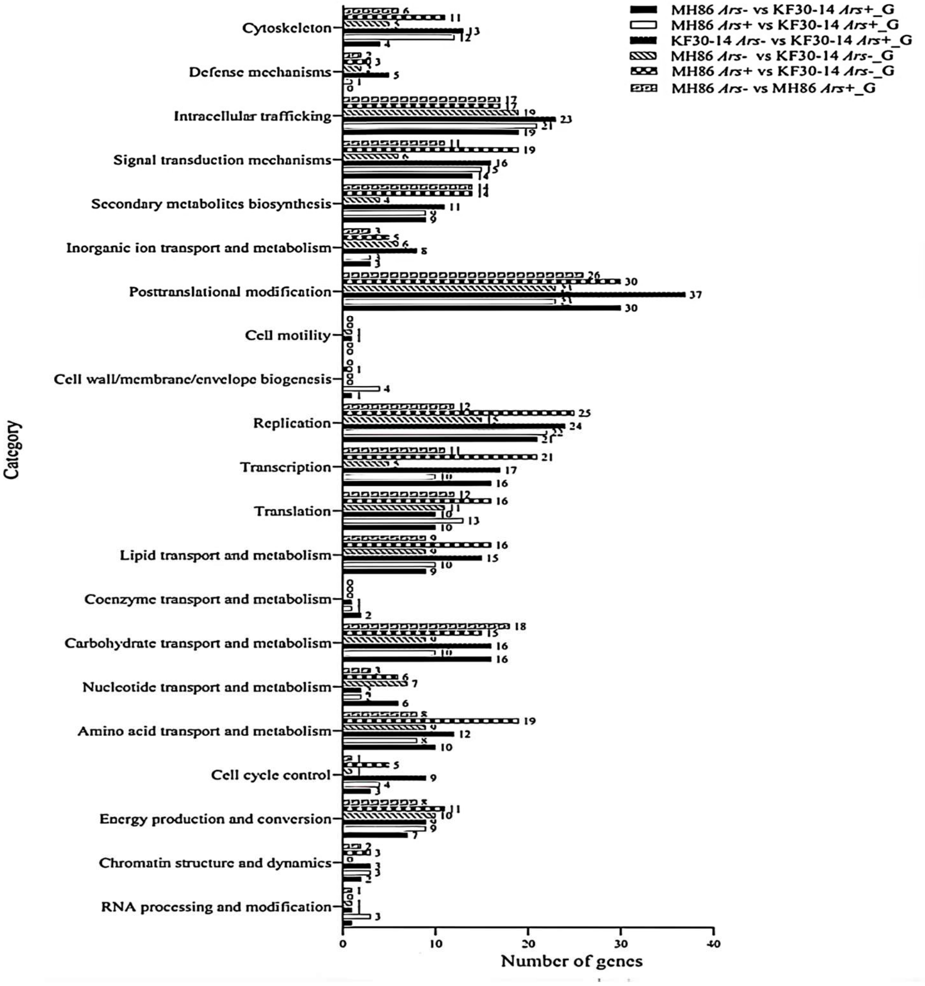

Figure S8: EggNOG Annotated classified statistical charts. The same description with table 4, 5

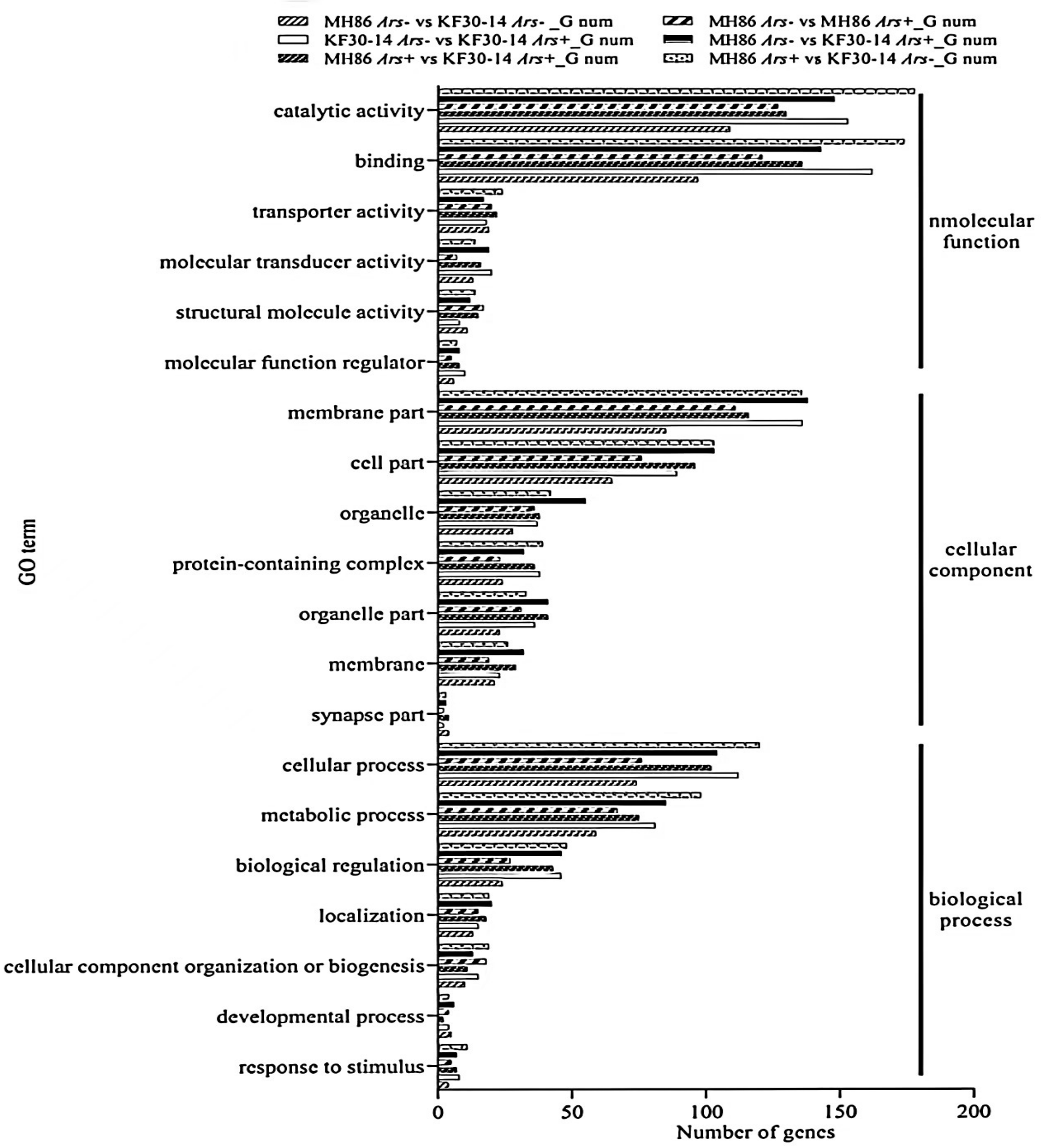

Figure S9: GO annotation analysis of DEGs.

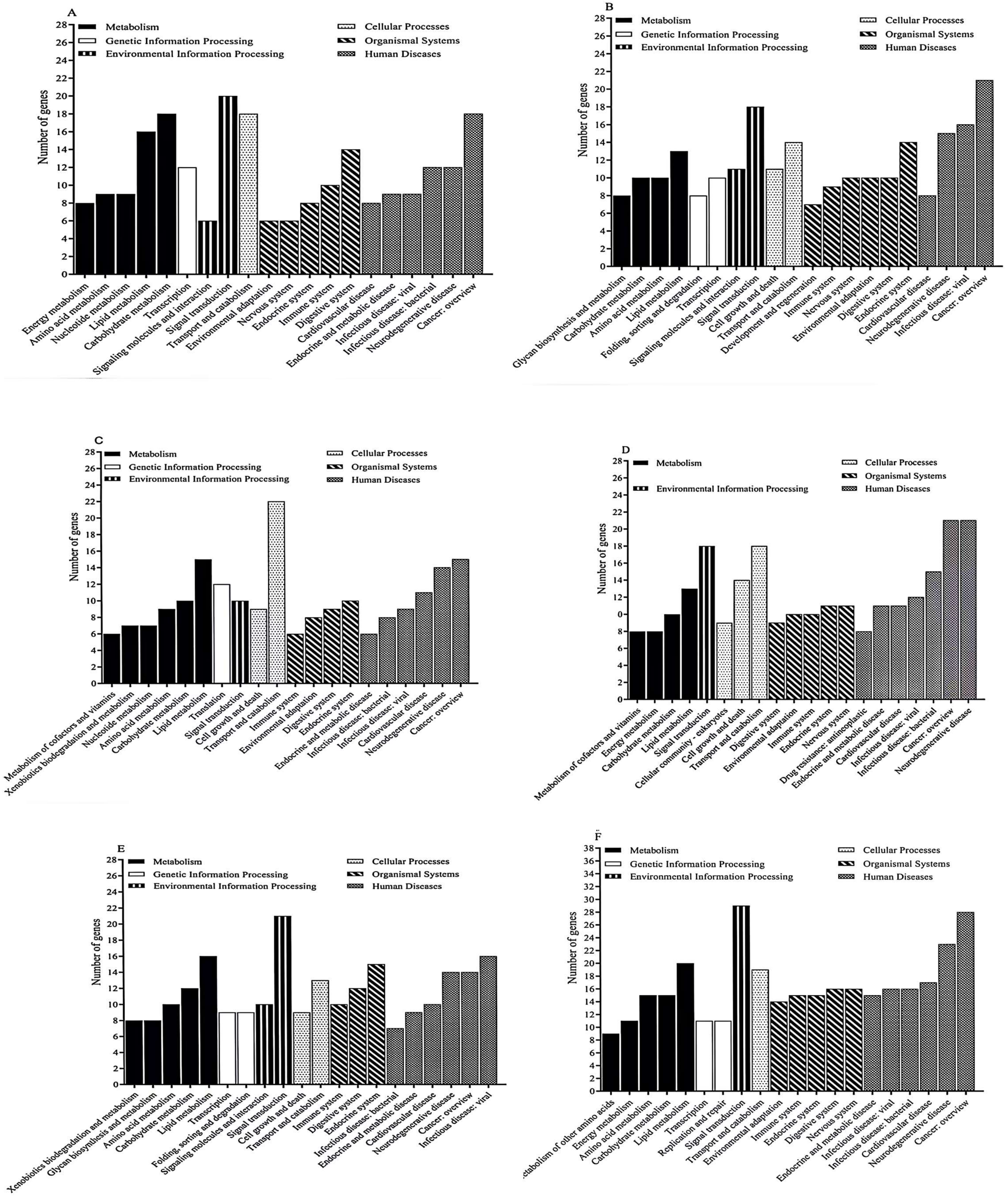

A
Metabolism
Genetic Information Processing
Environmental Information Processing
Cellular Processes
Organismal Systems
Human Diseases
Number of genes
Energy metabolism
Amino acid metabolism
Nucleotide metabolism
Lipid metabolism
Carbohydrate metabolism
Transcription
Signaling molecules and interaction
Signal transduction
Transport and catabolism
Environmental adaptation
Nervous system
Endocrine system
Immune system
Digestive system
Cardiovascular disease
Endocrine and metabolic disease
Infectious disease: viral
Infectious disease: bacterial
Neurodegenerative disease
Cancer: overview
B
Metabolism
Genetic Information Processing
Environmental Information Processing
Cellular Processes
Organismal Systems
Human Diseases
Number of genes
Glycan biosynthesis and metabolism
Carbohydrate metabolism
Amino acid metabolism
Lipid metabolism
Folding, sorting and degradation
Transcription
Signaling molecules and interaction
Signal transduction
Cell growth and death
Transport and catabolism
Development and regeneration
Immune system
Nervous system
Environmental adaptation
Digestive system
Endocrine system
Cardiovascular disease
Neurodegenerative disease
Infectious disease: viral
Cancer: overview
C
Metabolism
Genetic Information Processing
Environmental Information Processing
Cellular Processes
Organismal Systems
Human Diseases
Number of genes
Metabolism of cofactors and vitamins
Xenobiotics biodegradation and metabolism
Nucleotide metabolism
Amino acid metabolism
Carbohydrate metabolism
Lipid metabolism
Translation
Signal transduction
Cell growth and death
Transport and catabolism
Immune system
Environmental adaptation
Digestive system
Endocrine system
Endocrine and metabolic disease
Infectious disease: bacterial
Infectious disease: viral
Cardiovascular disease
Neurodegenerative disease
Cancer: overview
D
Metabolism
Environmental Information Processing
Cellular Processes
Organismal Systems
Human Diseases
Number of genes
Metabolism of cofactors and vitamins
Energy metabolism
Carbohydrate metabolism
Lipid metabolism
Signal transduction
Cellular community - eukaryotes
Cell growth and death
Transport and catabolism
Digestive system
Environmental adaptation
Immune system
Endocrine system
Nervous system
Drug resistance: antineoplastic
Endocrine and metabolic disease
Cardiovascular disease
Infectious disease: viral
Infectious disease: bacterial
Cancer: overview
Neurodegenerative disease
E
Metabolism
Genetic Information Processing
Environmental Information Processing
Cellular Processes
Organismal Systems
Human Diseases
Number of genes
Xenobiotics biodegradation and metabolism
Glycan biosynthesis and metabolism
Amino acid metabolism
Carbohydrate metabolism
Lipid metabolism
Transcription
Folding, sorting and degradation
Signaling molecules and interaction
Signal transduction
Cell growth and death
Transport and catabolism
Immune system
Digestive system
Endocrine system
Infectious disease: bacterial
Endocrine and metabolic disease
Cardiovascular disease
Neurodegenerative disease
Cancer: overview
Infectious disease: viral
F
Metabolism
Genetic Information Processing
Environmental Information Processing
Cellular Processes
Organismal Systems
Human Diseases
Number of genes
etabolism of other amino acids
Energy metabolism
Amino acid metabolism
Carbohydrate metabolism
Lipid metabolism
Transcription
Replication and repair
Signal transduction
Transport and catabolism
Environmental adaptation
Immune system
Endocrine system
Digestive system
Nervous system
Endocrine and metabolic disease
Infectious disease: viral
Infectious disease: bacterial
Cardiovascular disease
Neurodegenerative disease
Cancer: overview

Fig S10: KEGG annotation analysis. (A) the annotation analysis of DEGs in MH86 *Ars*-vs MH86 *Ars*$^{+}$; (B) the annotation analysis of DEGs in KF30-14 *Ars*$^{-}$ vs KF30-14 *Ars*$^{+}$; (C) the annotation analysis of DEGs in MH86 *Ars*$^{-}$ vs KF30-14 *Ars*$^{-}$; (D) the annotation analysis of DEGs in MH86 *Ars*$^{+}$ vs KF30-14 *Ars*$^{+}$; (E) the annotation analysis of DEGs in MH86 *Ars*$^{-}$ vs KF30-14 *Ars*$^{+}$; (F) the annotation analysis of DEGs in MH86 *Ars*$^{+}$ vs KF30-14 *Ars*$^{-}$.

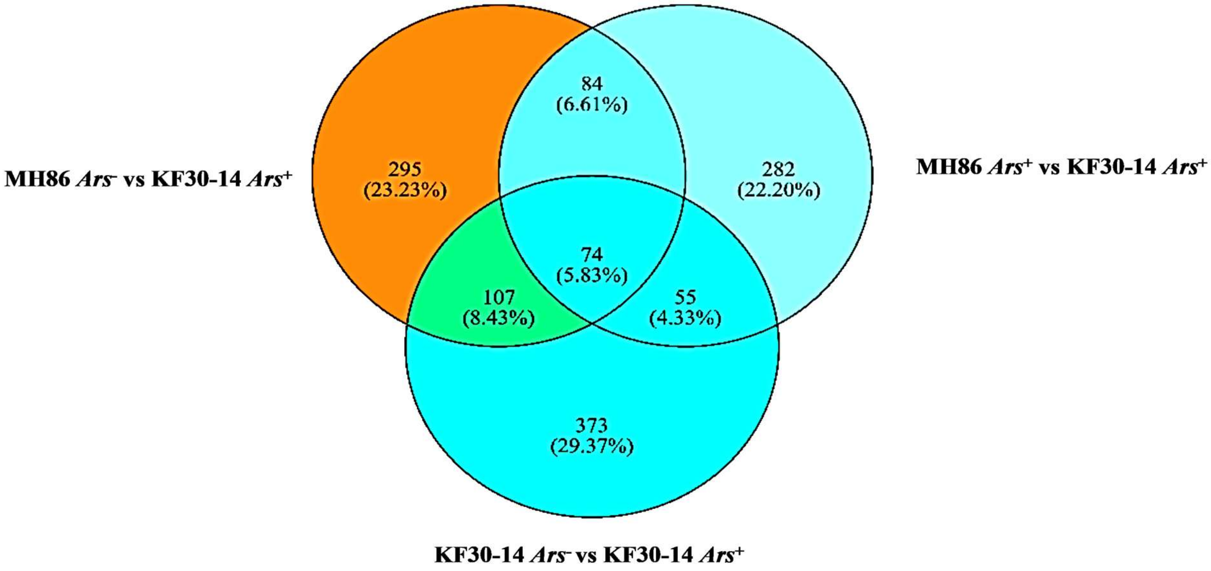

Figure S11: Venn diagram of differentially expressed genes between treatment groups.

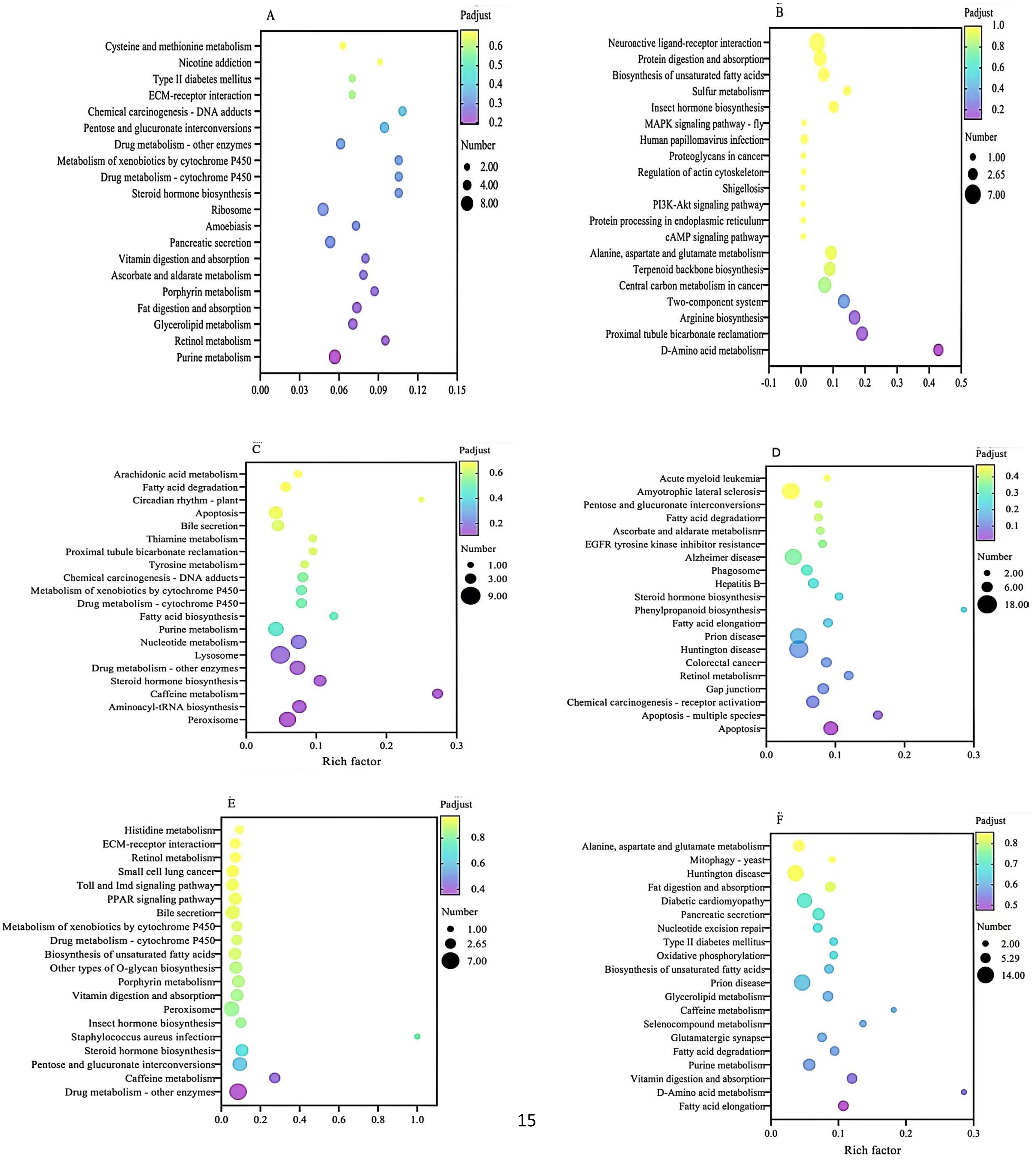
A
Padjust
0.6
0.5
0.4
0.3
0.2
Number
2.00
4.00
8.00
Cysteine and methionine metabolism
Nicotine addiction
Type II diabetes mellitus
ECM-receptor interaction
Chemical carcinogenesis - DNA adducts
Pentose and glucuronate interconversions
Drug metabolism - other enzymes
Metabolism of xenobiotics by cytochrome P450
Drug metabolism - cytochrome P450
Steroid hormone biosynthesis
Ribosome
Amoebiasis
Pancreatic secretion
Vitamin digestion and absorption
Ascorbate and aldarate metabolism
Porphyrin metabolism
Fat digestion and absorption
Glycerolipid metabolism
Retinol metabolism
Purine metabolism
0.00
0.03
0.06
0.09
0.12
0.15
B
Padjust
1.0
0.8
0.6
0.4
0.2
Number
1.00
2.65
7.00
Neuroactive ligand-receptor interaction
Protein digestion and absorption
Biosynthesis of unsaturated fatty acids
Sulfur metabolism
Insect hormone biosynthesis
MAPK signaling pathway - fly
Human papillomavirus infection
Proteoglycans in cancer
Regulation of actin cytoskeleton
Shigellosis
PI3K-Akt signaling pathway
Protein processing in endoplasmic reticulum
cAMP signaling pathway
Alanine, aspartate and glutamate metabolism
Terpenoid backbone biosynthesis
Central carbon metabolism in cancer
Two-component system
Arginine biosynthesis
Proximal tubule bicarbonate reclamation
D-Amino acid metabolism
-0.1
0.0
0.1
0.2
0.3
0.4
0.5
C
Padjust
0.6
0.4
0.2
Number
1.00
3.00
9.00
Arachidonic acid metabolism
Fatty acid degradation
Circadian rhythm - plant
Apoptosis
Bile secretion
Thiamine metabolism
Proximal tubule bicarbonate reclamation
Tyrosine metabolism
Chemical carcinogenesis - DNA adducts
Metabolism of xenobiotics by cytochrome P450
Drug metabolism - cytochrome P450
Fatty acid biosynthesis
Purine metabolism
Nucleotide metabolism
Lysosome
Drug metabolism - other enzymes
Steroid hormone biosynthesis
Caffeine metabolism
Aminoacyl-tRNA biosynthesis
Peroxisome
0.0
0.1
0.2
0.3
Rich factor
D
Padjust
0.4
0.3
0.2
0.1
Number
2.00
6.00
18.00
Acute myeloid leukemia
Amyotrophic lateral sclerosis
Pentose and glucuronate interconversions
Fatty acid degradation
Ascorbate and aldarate metabolism
EGFR tyrosine kinase inhibitor resistance
Alzheimer disease
Phagosome
Hepatitis B
Steroid hormone biosynthesis
Phenylpropanoid biosynthesis
Fatty acid elongation
Prion disease
Huntington disease
Colorectal cancer
Retinol metabolism
Gap junction
Chemical carcinogenesis - receptor activation
Apoptosis - multiple species
Apoptosis
0.0
0.1
0.2
0.3
E
Padjust
0.8
0.6
0.4
Number
1.00
2.65
7.00
Histidine metabolism
ECM-receptor interaction
Retinol metabolism
Small cell lung cancer
Toll and Imd signaling pathway
PPAR signaling pathway
Bile secretion
Metabolism of xenobiotics by cytochrome P450
Drug metabolism - cytochrome P450
Biosynthesis of unsaturated fatty acids
Other types of O-glycan biosynthesis
Porphyrin metabolism
Vitamin digestion and absorption
Peroxisome
Insect hormone biosynthesis
Staphylococcus aureus infection
Steroid hormone biosynthesis
Pentose and glucuronate interconversions
Caffeine metabolism
Drug metabolism - other enzymes
0.0
0.2
0.4
0.6
0.8
1.0
F
Padjust
0.8
0.7
0.6
0.5
Number
2.00
5.29
14.00
Alanine, aspartate and glutamate metabolism
Mitophagy - yeast
Huntington disease
Fat digestion and absorption
Diabetic cardiomyopathy
Pancreatic secretion
Nucleotide excision repair
Type II diabetes mellitus
Oxidative phosphorylation
Biosynthesis of unsaturated fatty acids
Prion disease
Glycerolipid metabolism
Caffeine metabolism
Selenocompound metabolism
Glutamatergic synapse
Fatty acid degradation
Purine metabolism
Vitamin digestion and absorption
D-Amino acid metabolism
Fatty acid elongation
0.0
0.1
0.2
0.3
Rich factor

Figure S12: KEGG enrichment analysis of DEGs in each treatment comparison. (A) the enrichment results of DEGs in MH86 *Ars*$^{-}$vs MH86 *Ars*$^{+}$; (B) the enrichment results of DEGs in KF30-14 *Ars*$^{-}$vs KF30-14 *Ars*$^{+}$; (C) the enrichment results of DEGs in MH86 *Ars*$^{-}$ vs KF30-14 *Ars*$^{-}$; (D) the enrichment results of DEGs in MH86 *Ars*$^{+}$ vs KF30-14 *Ars*$^{+}$; (E) the enrichment results of DEGs in MH86 *Ars*$^{-}$ vs KF30-14 *Ars*$^{+}$; (F) the enrichment results of DEGs in MH86 *Ars*$^{+}$ vs KF30-14 *Ars*$^{-}$.
